# Towards genomic database of Alexander disease to identify variations modifying disease phenotype

**DOI:** 10.1038/s41598-019-51390-8

**Published:** 2019-10-14

**Authors:** Rei Yasuda, Masakazu Nakano, Tomokatsu Yoshida, Ryuichi Sato, Hiroko Adachi, Yuichi Tokuda, Ikuko Mizuta, Kozo Saito, Jun Matsuura, Masanori Nakagawa, Kei Tashiro, Toshiki Mizuno

**Affiliations:** 1 Department of Neurology, Graduate School of Medical Science, Kyoto Prefectural University of Medicine, Kyoto, Japan; 2 Department of Genomic Medical Sciences, Kyoto Prefectural University of Medicine, Kyoto, Japan; 3 Department of Neurology, North Medical Center, Kyoto Prefectural University of Medicine, Kyoto, Japan

**Keywords:** Disease genetics, Neurodegeneration

## Abstract

Alexander disease (AxD) is an extremely rare neurodegenerative disorder caused by glial fibrillary acidic protein (GFAP) gene mutations. Compared with the cerebral type, which is characterized by infantile onset, the bulbospinal type and intermediate form are associated with a late onset, spanning from juveniles to the elderly, and more diverse clinical spectrum, suggesting the existence of factors contributing to phenotypic diversity. To build a foundation for future genetic studies of this rare disease, we obtained genomic data by whole exome-sequencing (WES) and DNA microarray derived from thirty-one AxD patients with the bulbospinal type and intermediate form. Using this data, we aimed to identify genetic variations determining the age at onset (AAO) of AxD. As a result, WES- or microarray-based association studies between younger (<45 years; n = 13)- and older (≥45 years; n = 18)-onset patients considering the predicted GFAP-mutation pathogenicity identified no genome-wide significant variant. The candidate gene approach identified several variants likely correlated with AAO (p < 0.05): *GAN*, *SLC1A2*, *CASP3*, *HDACs*, and *PI3K*. Although we need to replicate the results using an independent population, this is the first step towards constructing a database, which may serve as an important tool to advance our understanding of AxD.

## Introduction

Alexander disease (AxD, OMIM #203450)^1^ is an extremely rare neurodegenerative disorder pathologically characterized by cytoplasmic inclusions called Rosenthal fibers in astrocytes. The prevalence of AxD is estimated to be one case per 2.7 million individuals^2^. Since mutations of glial fibrillary acidic protein (*GFAP*) were identified as the cause of AxD in 2001^3^, AxD patients with a wide spectrum of clinical presentations have been reported. AxD can be classified into three subtypes: cerebral type, bulbospinal type, and intermediate form, based on the neurological symptoms and lesion location. Cerebral type is characterized by an infantile or juvenile onset with frontal-dominant white matter abnormalities on brain MRI and certain neurological symptoms, including convulsions, macrocephaly, and psychomotor developmental delay. Bulbospinal type is characterized by a juvenile or adult onset with an abnormal signal or atrophy of the medulla oblongata and/or cervical cord on MRI. The neurological symptoms of bulbospinal type include muscle weakness, spasticity, and bulbar symptoms, which occur in various combinations. The intermediate form is characterized by juvenile or adult onset, and by the coexistence of neurological features of both the cerebral and bulbospinal types.

To date, more than 100 *GFAP* mutations have been reported to be associated with AxD (http://www.waisman.wisc.edu/alexander-disease/). A total of 70% of patients with the cerebral type have amino acid mutations located in either residue R79, R88, or R239^2^. On the contrary, a wide distribution of AAO and many mutations without hot spots have been reported in bulbospinal type and intermediate form. Moreover, an identical mutation, such as R416W, was reported in cerebral type, bulbospinal type, and also intermediate form^4^. These findings suggest the presence of some modifying factors leading to the phenotypic variation in the bulbospinal type and intermediate form in addition to GFAP mutations, while the almost constant phenotype of the cerebral type may be determined mostly by GFAP mutations themselves.

Recent genome-wide association studies on Huntington disease and Duchenne muscular dystrophy, both of which are monogenic neurological diseases, identified some susceptible loci as modifier genes that are likely to contribute to the diversity of AAO and age at loss of ambulation, respectively^5,6^. However, the literature on extremely rare diseases including AxD mainly comprises case reports from different institutes. An association study involving a number of AxD patients has not been reported to date.

We collected blood samples from and clinical information on AxD patients referred from facilities throughout Japan, and obtained genomic data from 31 bulbospinal-type and intermediate-form AxD patients by whole-exome sequencing (WES) and microarray analysis. Then, we performed association analyses focusing on AAO in an attempt to identify genes responsible for the phenotypic diversity of the bulbospinal type and intermediate form. Although no genome-wide significant variant was detected, we identified genetic variations likely to be associated with AAO by focusing on AxD-related genes.

## Results

### Clinical information on subjects

**Figure 1:**
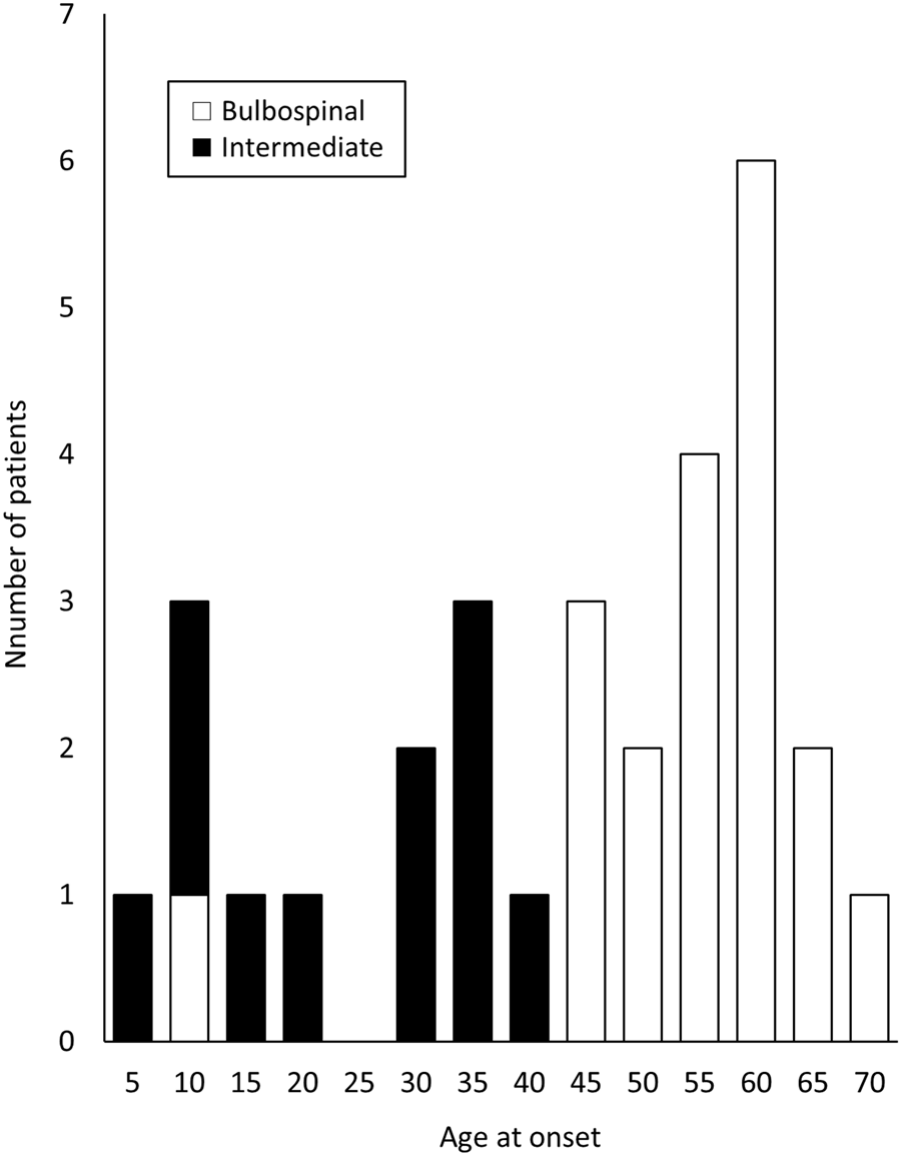
Distribution of age at onset of patients with Alexander disease. The age at onset (AAO) was distributed widely and bimodally: one peak was in those in their teens to thirties, and the other peak was at around 60 years. Most of the younger-onset patients presented with intermediate form, whereas older-onset patients presented with bulbospinal type. The boundary of both AAO and the phenotype was around 45 years. White and black bars indicate bulbospinal type and intermediate form, respectively.

Clinical information is presented in Table 1. Twenty-five patients had already been reported in the literature^7–17^. The distribution of AAO and clinical classification (bulbospinal type or intermediate form) are shown in Fig. 1. The AAO was distributed widely (5 to 72 years) and bimodally: one peak was in those in their teens to thirties and the other peak was around 60 years, and the boundary was approximately 45 years. Thus, we defined the subjects with an onset at younger than 45 years as a “younger-onset” group (n = 13) and those with an onset at 45 years or older as an “older-onset” group (n = 18). Although the exact AAO of patient 12 was unknown, she was included in the younger-onset group because she had been diagnosed at 32 years. All patients in the older-onset group had the bulbospinal type, whereas most of the younger-onset patients had intermediate form (92%). Gait disturbance was the most frequent initial symptom in both the younger (62%)- and older (33%)-onset patients. In the older-onset patients, bulbar symptoms such as dysarthria and dysphagia were the second most frequent initial symptoms (22%).

**Table 1:** Clinical characteristics of the patients.

No.	*GFAP* mutation	Sex	Age at diagnosis, years	Age at onset, years	Initial symptom	Ref
1	R79H	F	51	5	Gait disturbance	^7^
2^a^	R126_L127dup	M	12	10	Gait disturbance	^8^
3	D360N	F	36	14	Dysarthria	
4^a^	L357P	F	24	18	Gait disturbance	^8^
5	E243dup	F	27	22	Blurred vision	^9^
6	A268D	M	47	30	Gait disturbance	^8^
7	A244V	F	54	32	NA	^8^
8	R79H	M	37	36	Gait disturbance	^7, 10, 11^
9	R79H	F	40	38	Dementia	^7, 12^
10^a^	R416W	M	39	38	Gait disturbance	^8^
11^a^	Y242N	F	65	40	Gait disturbance	^8, 13^
12	R276L	F	32	NA	Mental retardation	
**Bulbospinal type**						
13	E362G	M	58	12	Gait disturbance	^7^
14	R416W	M	49	45	Limb weakness	^8^
15	L123P	F	51	45	Weakness	^8^
16	R124_L125insE	F	47	46	Gait disturbance	^8, 14^
17	M74T	M	56	51	Limb clumsiness	^7, 10, 15^
18	G301D	F	52	51	Gait disturbance	^8^
19	R258C	M	59	55	Gait disturbance	
20	M74T	M	60	56	Limb weakness	
21	E210K	F	65	58	Incontinence	^8, 16^
22	N386S	M	60	59	Dysarthria	^8^
23	R258H	M	65	60	Limb weakness	^8, 16^
24	N386S	M	68	61	NA	
25	N386S	M	65	62	Gait disturbance	^8, 16^
26	N386S	M	66	63	Dysarthria	^8^
27	R70W	M	67	64	Dysphagia	^7, 10^
28	R70W	F	65	64	Dysarthria	^8, 16^
29	R70W	M	66	65	Limb weakness	
30	N102K	F	71	66	Gait disturbance	^8^
31	N386S	M	72	72	Gait disturbance	^17^

^a^These patients had been diagnosed with the bulbospinal type^8^, but they were subsequently classified with the intermediate form based on strict re-evaluation in this study.

Ref = reference; F = female; M = male; NA = not available. Phenotypic differences in patients with the same genotype may be due to genetic modifiers.

### *GFAP* genotype- phenotype correlation

**Figure 2:**
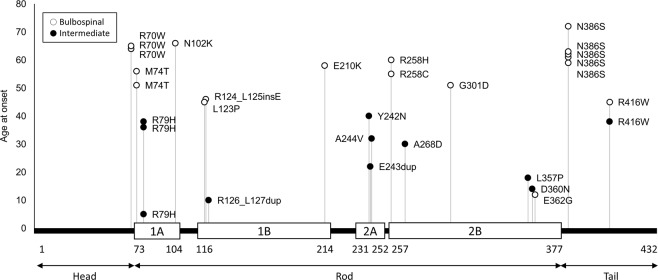
Schematic localization of *GFAP* mutation and age at onset. Age at onset (longitudinal axis) and amino acid number of *GFAP* mutations (horizontal axis) of each patient are plotted. White and black dots indicate bulbospinal Alexander disease and intermediate form, respectively. GFAP protein consists of head, rod, and tail domains. The rod domain includes four alpha-helical subdomains (white boxes).

**Figure 3:**
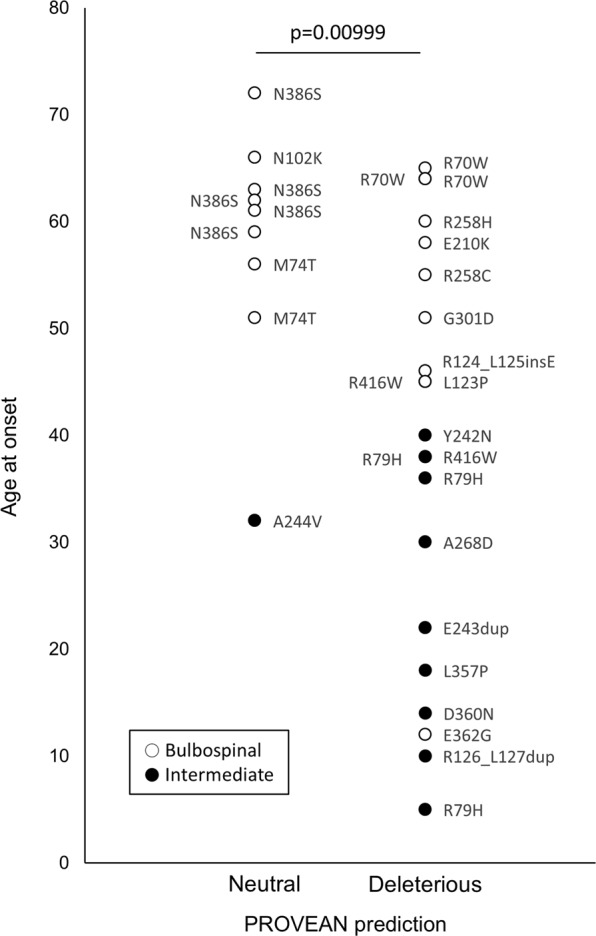
The predicted pathogenicity of *GFAP* mutations and age at onset. The pathogenicity of *GFAP* mutations was divided into “Neutral” and “Deleterious” using the prediction tool PROVEAN. The mean age at onset of the “Deleterious” group (38.9 ± 8.8 years) was significantly younger than that of the “Neutral” group (58.0 ± 8.8 years) (*p* = 0.00999, t-test). White and black dots indicate bulbospinal Alexander disease and intermediate form, respectively.

A total of 21 unique *GFAP* mutations were identified in the 31 patients. They were distributed widely including head, rod, and tail domains, without hot spots (Fig. 2). As for 5 mutations shared by multiple patients, patients with R70W, M74T, and N386S all belonged to the older-onset group, and those with R79H belonged to the younger-onset group, whereas those with R416W were distributed in both younger- and older-onset groups. The remaining sixteen mutations were singletons. These findings suggested the difficulty of analyzing genotype-phenotype correlations for each mutation. Alternatively, we divided the mutations into two groups: “Neutral” and “Deleterious”, based on the prediction tool PROVEAN and compared AAO between the two groups (Fig. 3). Nine patients had “Neutral” and 22 patients had “Deleterious” mutations. The mean AAO was significantly different between “Neutral” (58.0 ± 8.8 years) and “Deleterious” (38.9 ± 8.8 years) groups (*p* < 0.01, t-test).

### WES/Microarray analysis

WES was performed to identify exonic functional variants, and microarray was used for genome-wide analysis. In WES, the mean depth (defined as “total number of reads mapped to the target regions/total number of target regions”) was 169, and the mean coverage (defined as “number of target regions covered over 50 reads/total number of target regions”) was 90.7%. In microarray analysis, the average call rate was 99.8%.

**Figure 4:**
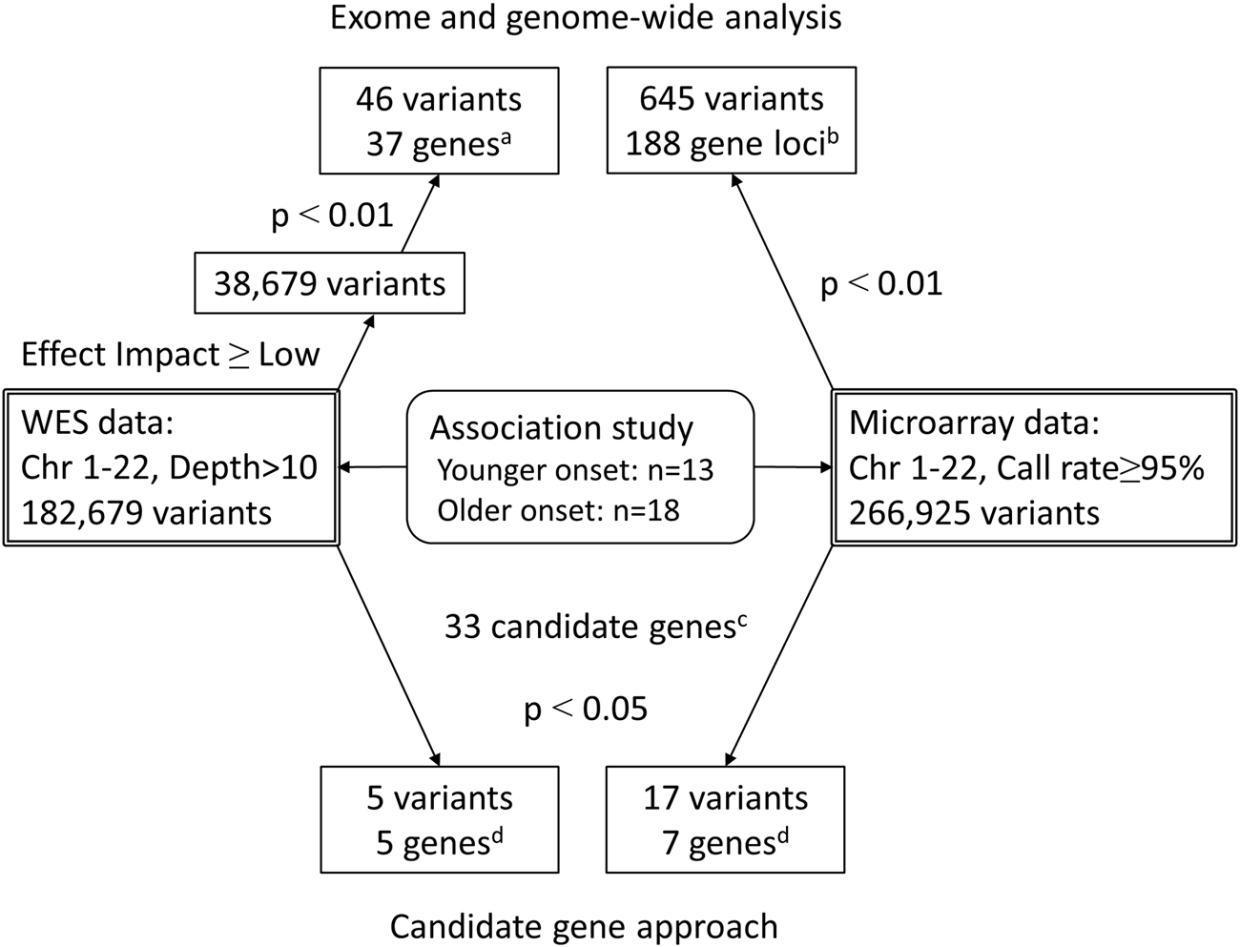
Flowchart of association analysis. We performed WES- and microarray-based association analysis (upper) and also adopted a candidate gene approach (lower). Initially, an association study between younger- and older-onset patients considering predicted *GFAP* mutation pathogenicity was conducted. There was no variant reaching genome-wide significance. Next, we focused on variants in candidate genes related to the pathophysiology of Alexander disease. (**a**–**d**) Lists of the variants or genes are presented in Supplementary Tables S1(a), S2(b), S5(c) and 2(d). WES = whole-exome sequencing; Chr = chromosome.

An association study with logistic regression was performed by considering the predicted pathogenicity of the *GFAP* mutations described above. The number of variants investigated in WES and microarray analyses were 38,679 and 266,925, respectively (Fig. 4). Forty-six variants (37 genes) had a *p*-value < 0.01 in WES (Supplementary Table S1) and 645 variants (188 gene loci) met this value in microarray analysis (Supplementary Table S2). Of the forty-six variants with a *p*-value < 0.01 in WES data, thirty-five variants (76%) were reproducible in microarray data. The *p*-value of the recessive model tended to be unavailable or high mainly due to the absence or too small a number of minor allele homozygotes. No variant had a *p*-value below the significance level after Bonferroni correction using either analysis (WES-based significance: *p* < 1.29 × 10^−6^, microarray-based significance: *p* < 1.87 × 10^−7^).

We additionally searched for modifiers solely in 19 patients with mutations that cause the bulbospinal type, because their symptoms are comparatively mild and the effect of these *GFAP* mutations might not be severe (Supplementary Materials S3 and S4). Sixteen and 22 variants showed *p*-values below 1.0 × 10^−5^ in WES and microarray analysis, respectively. One variant, p.Ala155Ala in *TRABD2B*, reached WES-based significance (*p* = 2.05 × 10^−7^). However, after excluding the outlier with a markedly early age at onset, no variant showed *p*-values below 1.0 × 10^−5^.

### Candidate gene approach

The lack of WES- or microarray-based significant variants may be expected due to the small sample size and weaker modifier effect than *GFAP* mutations. To identify likely associated variants in AxD-related genes, we employed a candidate gene approach. Among 33 genes reported to have an association with AxD in the literature (Supplementary Table S5), the variants of 5 genes had a *p*-value < 0.05 using next-generation sequencing data (Table 2): upstream insertion variant of *HDAC4*; intron single nucleotide polymorphisms (SNPs) of *CASP3* and *GAN*; and missense variants of *PIK3CG* and *PIK3C2G*. The variants of 7 genes were identified with a *p*-value < 0.05 using microarray data (Table 2): intron SNPs of *HDAC9*, *PIK3AP1*, *SLC1A2*, *PIK3C2G*, *GAN* and *PIK3R5*; 3’UTR SNP of *HDAC9*; and missense variants of *PIK3CG* and *PIK3C2G*. The missense variants in *PIK3CG* and *PIK3C2G* were identified with a *p*-value < 0.05 using both next-generation sequencing and microarray data.

**Table 2:** Summary of next-generation sequencing and microarray candidate gene analysis in association with age at onset.

Chr	Position	Minor/ Major Allele	MAF	Location (Effect)	Gene Name	Genotype^a^		*p*-value	Odds Ratio^b^
						Younger Onset	Older Onset		
**Next-generation sequencing**									
2	240323905	T/TG	0.27	upstream	*HDAC4*	0/1/12	1/11/6	0.018	0.06
4	185559487	G/C	0.21	intron	*CASP3*	0/1/12	0/8/10	0.009	0.04
7	106509331	A/C	0.15	exon (missense)	*PIK3CG*	0/7/6	0/3/15	0.033	11.94
12	18649057	T/C	0.31	exon (missense)	*PIK3C2G*	0/5/8	1/13/4	0.030	0.14
16	81398520	G/A	0.29	intron	*GAN*	0/8/5	0/2/16	0.014	18.22
**Microarray**									
7	18328833	C/T	0.18	intron	*HDAC9*	1/0/12	0/9/9	0.035	0.08
7	18979516	G/A	0.27	intron	*HDAC9*	2/7/4	0/6/12	0.031	5.26
7	19035920	G/A	0.39	3’ UTR	*HDAC9*	0/4/9	4/12/2	0.008	0.07
7	106509331	A/C	0.16	exon (missense)	*PIK3CG*	0/7/6	0/3/15	0.033	11.94
10	98395083	T/C	0.44	intron	*PIK3AP1*	0/6/7	7/7/4	0.022	0.17
10	98403893	A/G	0.45	intron	*PIK3AP1*	4/7/2	2/9/7	0.046	5.14
11	35399110	A/G	0.23	intron	*SLC1A2*	1/9/3	0/3/15	0.009	11.87
12	18641138	A/G	0.32	intron	*PIK3C2G*	0/5/8	1/13/4	0.030	0.14
12	18649057	T/C	0.32	exon (missense)	*PIK3C2G*	0/5/8	1/13/4	0.030	0.14
12	18651702	T/C	0.39	intron	*PIK3C2G*	1/4/8	3/12/3	0.018	0.06
12	18787403	G/A	0.37	intron	*PIK3C2G*	1/9/3	3/6/9	0.036	7.28
16	81361294	A/C	0.31	intron	*GAN*	3/7/3	0/6/12	0.029	5.59
16	81363643	C/A	0.42	intron	*GAN*	6/6/1	0/8/10	0.014	15.30
16	81372725	A/C	0.32	intron	*GAN*	4/6/3	0/6/12	0.024	5.56
16	81395603	T/C	0.40	intron	*GAN*	6/6/1	0/7/11	0.011	17.61
16	81397804	T/C	0.16	intron	*GAN*	0/8/5	0/2/16	0.014	18.22
17	8834595	A/G	0.45	intron	*PIK3R5*	3/9/1	3/7/8	0.020	16.98

The variants with a *p*-value < 0.05 in either additive, dominant, or recessive models are shown.

^a^The number of patients with minor allele homozygous/heterozygous/major allele homozygous is shown.

^b^Odds ratio represents the odds of a younger onset with the minor compared with major allele.

Chr = chromosome; MAF = minor allele frequency; UTR = untranslated region.

These results were verified by Sanger sequencing of the SNP with the lowest *p*-value in each gene (Supplementary Fig. S6–1). In all patients, genotypes of the 9 representative SNPs determined by WES/microarray were consistent with those determined by the Sanger method, except for *PIK3C2G* SNP (position chr12:18651702, rs2305220) in one patient. In this particular patient, rs2305220 was T/T based on microarray; however, it was T/C based on Sanger sequencing. This discrepancy may have been due to another heterozygous SNP, rs191094996, located next to rs2305220 (Supplementary Fig. S6−2), suggesting a genotyping error caused by mismatched hybridization in microarray. When using corrected genotyping data, the *p*-value of *PIK3C2G* rs2305220 remained at < 0.05 (*p* = 0.0151).

## Discussion

Our study is the first genome-wide analysis focusing on genetic modifiers in extremely rare monogenic diseases including AxD. The predicted pathogenicity of *GFAP* mutation was significantly correlated with AAO. In the association study considering *GFAP* mutation pathogenicity, we detected several genes likely to be associated with AAO, which may contribute to the pathophysiology of AxD, although no genome-wide variants with significance after Bonferroni correction were detected.

In this study, we obtained clinical information linked with genomic data for the first time for AxD, being helpful to elucidate phenotype-genotype associations. Because of the extreme rarity of AxD, independent patients in different hospitals were recruited. To standardize the format of their clinical data, we used a questionnaire including AAO, initial symptoms, family history, neurological examination, brain/spine MRI findings, physiological testing, and degree of disability. Furthermore, we re-evaluated original MRI data based on unified standards.

In our study analyzing patients with bulbospinal type and intermediate form of AxD, AAO was distributed widely from 5 to 72 years, and older-onset patients (AAO ≥ 45) accounted for 58%, which is a very large number of older-onset patients compared with a previous study where the mean AAO was around 21 years old^18^. That is, AxD, which belongs to monogenic neurological diseases, has a broader AAO than previously thought, suggesting the existence of modifier genes other than causative *GFAP* mutations contributing to the diversity of AAO, especially in the bulbospinal type and intermediate form. We also demonstrated that the *GFAP* mutation pathogenicity predicted by sequence conservation was significantly correlated with AAO. The mean AAO was 20 years younger in patients with “Deleterious” compared with “Neutral” mutations. We applied this finding to association analysis.

In the WES- or microarray-wide association study, each variant was analyzed considering the predicted pathogenicity of *GFAP* mutations as a covariate. A WES-based association study is a novel approach, validated with microarray-based analysis. A total of 76% of the variants based on the WES-based association study with a *p*-value < 0.01 were reproducible in microarray-based analysis, suggesting the reliability of the WES-based association study. Such a WES-based association study is expensive compared with microarray analysis, but it has the merit of identifying functional variants directly. A WES-based association study would be particularly useful if WES data already exist.

We could not detect any variants reaching WES- or microarray-based significance. This is mainly due to not only the small sample size but also the effect of *GFAP* mutations. Intermediate-type patients showed a more severe phenotype and a high frequency of *GFAP* mutations predicted as deleterious (11 out of 12, 92%). On the other hand, bulbospinal-type patients showed a mild phenotype and their *GFAP* mutations included both deleterious (11 out of 19, 58%) and neutral (8 out of 19, 42%) forms. These results suggest the weaker effect of *GFAP* mutations in the bulbospinal type. In the quantitative trait locus (QTL) analysis of bulbospinal-type patients, although the influence of one outlier was not negligible, comparatively lower *p*-values were obtained. Increasing the number of patients, especially with the bulbospinal type, will be critical to identify genetic modifiers in the future.

In rare monogenic disorders, a small number of samples will lead to a weak statistical power, but it is rational to focus on the pathophysiology related to causative genes playing a primary role. With a focus on variants in candidate genes selected from the literature, five significant candidate genes with *p*-values under 0.05 were identified. *GAN* encodes gigaxonin which plays a role in the neurofilament architecture. Gigaxonin is mutated in giant axon neuropathy (OMIM #256850), in which abnormal GFAP aggregation occurs. A recent report indicated that gigaxonin targets GFAP for proteasomal degradation^19^. Interestingly, the decreased proteasomal function in astrocytes would contribute to increase the toxic effects of mutant GFAPs^20^, suggesting that the dysfunction of gigaxonin may be involved in accelerating GFAP aggregation. *SLC1A2* encodes the major glutamate transporter of astrocytes, GLT-1^21^. Reductions of GLT-1 protein and mRNA were exacerbated in AxD mouse models^22^. The alteration of GLT-1 expression may affect the susceptibility to glutamate excitotoxity and neuron death. *CASP3* encodes a protease, caspase 3, which plays a central role in cell apoptosis. Caspase 3 activation was reported to be correlated with mutant GFAPs in the C-terminal domain of GFAP, leading to the loss of astrocyte viability^23^. *HDACs* encode histone deacetylases, which play a role in the regulation of gene expression. Some HDACs were reported to control GFAP expression in primary human astrocytes^24^. In an exome next-generation sequencing study of two half-siblings with adult-onset AxD, the *HDAC6* variant was shown to modulate a motor neuron disease-like phenotype^25^. *PI3K* encodes phosphatidylinositol-4,5-bisphosphate-3-kinase that is upstream of the key events leading to GFAP expression^26^, suggesting that the dysregulation of *GFAP* expression due to *PI3K* mutations may influence an abnormal GFAP aggregation process. Mutations in components of the PI3K-AKT pathway were found in patients with megalencephaly^27^, which is one of the major symptoms of intermediate form.

We validated the results of the candidate gene approach by Sanger sequencing to rule out latent genotype errors caused by WES/microarray methods. Surprisingly, ~5% errors caused by NGS in cancer samples were reported^28^. Of 273 genotyping data (9 variants of 31 patients) by Sanger sequencing, one genotype mismatched with that determined by microarray. This was probably due to another adjacent variant identified by the Sanger method. We should verify genotyping data by the Sanger method, especially when positive results are obtained by WES/microarray.

Recently, research involving a *Drosophila* AxD model identified a number of modifier genes^29^. Interestingly, of the 188 genes (*p*-value < 0.01) identified by our microarray analysis (Fig. 4), 9 genes (*ABAT*, *MYCBP2*, *OPCML*, *PRKG1*, *TRIO*, *CDKAL1*, *CACNA2D3*, *GUCY1A2*, and *GPC5*) corresponded to the modifier genes identified in the *Drosophila* model^29^.

Our study has several limitations. First, because the patients were all Japanese, our results may not be applicable to other ethnicities. Second, the number of patients was too small to detect variants with a weak effect. It was difficult to collect an adequate number of patients due to the markedly low prevalence of AxD: one case per 2.7 million individuals, of which bulbospinal type and intermediate form were estimated to collectively comprise 65%^2^. Finally, we could not collect a sufficient number of patients with identical *GFAP* mutations. Alternatively, we applied the *in silico* prediction of *GFAP* mutation pathogenicity. Of the prediction tools based on phylogenic conservation such as PROVEAN, PolyPhen-2, SIFT, and Mutation Assessor, we selected PROVEAN because it covers insertion/deletion mutations. To improve the prediction of pathogenicity, effects of mutations on the structure or stability of GFAP protein and annotations other than conservation should be considered. Combined Annotation–Dependent Depletion (CADD) is a prediction method to objectively integrate many diverse annotations into a single measure^30^. We compared predictions by PROVEAN, PolyPhen-2, SIFT, Mutation Assessor, and CADD (Supplementary Table S7). For some mutations, predictions were not consistent among the tools. In addition, we considered predicted pathogenicity based on the protein structure or stability reported previously^31,32^ (Supplementary Table S7). These findings suggest that prediction method used in this study is not definite. The use of multiple prediction tools and consideration of the protein structure or stability may be necessary in future analyses. To overcome these limitations in the future, recruiting larger numbers of AxD patients is indispensable. The identification of significant genome-wide genes will help elucidate the pathophysiology and therapeutic targets of AxD.

In conclusion, we conducted genome-wide analyses for the first time in AxD, which is an extremely rare monogenic disease, by employing two methods: WES and microarray. The association study in consideration of *GFAP* pathogenicity identified several variants of candidate genes with a *p*-value < 0.05. The results of the WES-based association study were comparable to those generated by microarray-based analysis. It is challenging to identify modifier genes with genome-wide significance for extremely rare monogenic disorders. Efforts toward establishing a genomic database of AxD may be important for confirming the results of the present study as well as identifying other potential modifier genes.

## Methods

### Participants

Forty-two Japanese AxD patients of 40 families with heterozygous *GFAP* mutations were diagnosed between 2004 and 2016, all of whom were referred to our facility from other hospitals throughout Japan for *GFAP* gene analysis because of suspected AxD. Clinical information, including AAO, family history, initial symptom, neurological findings, and MRI data, were obtained by survey questionnaire form. To strictly classify the clinical types of patients, brain and spinal MRI findings were re-evaluated by experienced neurologists (RY, TY) using original image data. Four patients who gave written informed consent for genetic testing, but did not consent to participate in further research, were excluded from this study. From the 38 patients who gave written consent of this study, we excluded five patients with cerebral type because the history and prognosis of cerebral type are almost constant for each case, and thus AAO of this form may be almost exclusively determined by *GFAP* mutations. Of the remaining 33 patients from 31 families, who met the diagnostic criteria of bulbospinal type or intermediate form, 31 probands were included. All participants provided written informed consent. This study was performed in accordance with the declaration of Helsinki and approved by the Ethical Review Board of Kyoto Prefectural University of Medicine.

### WES and microarray analysis

We extracted genomic DNA from leukocytes and performed WES using standard protocols. DNA was captured with SureSelect Human All Exon V5 (Agilent Technologies, Santa Clara, CA, USA) and sequenced using Illumina HiScanSQ (Illumina, San Diego, CA, USA) with 108-bp paired-end reads in the NGS Core Facility at Kyoto Prefectural University of Medicine. After base calling with CASAVA v.1.8.2 (Illumina), reads were aligned to the human reference genome sequence (GRCh37/hg19) with Burrows-Wheeler Aligner v.0.7.12^33^. PCR duplicates were removed with Piacard v1.119. Single nucleotide variants and indels were called with the Genome Analysis Toolkit UnifiedGenotyper v.1.6-13^34^. Called variants were annotated with SnpEff v.4.0e^35^. Autosomal variants whose effect-impact was predicted as “High”, “Moderate”, or “Low” (except for synonymous variants) were analyzed. The bases without variant calls were considered to be identical genotypes to the reference allele. To ensure the accuracy of genotyping, variants with a minimum depth of 10 or lower were excluded.

In microarray analysis, we genotyped 551,839 genetic markers with InfiniumCoreExome-24 v.1.1 BeadChip (Illumina) and Genome Studio Software v.2011.1 (Illumina) according to the manufacturer’s instructions. The genotype data were obtained by using Genome Studio Software v.2011.1 (Illumina). A quality control filter was applied as: minor allele frequency >0 (i.e., monomorphic markers were excluded) and a call rate ≥95% for autosomal markers.

To validate the accuracy of WES/microarray genotyping, the SNP with the lowest *p*-value for each significant candidate gene was chosen and further PCR and Sanger sequencing were performed. The data were analyzed by Sequencher 4.10.1 (Gene Codes Corporation, Ann Arbor, MI, USA). The PCR/sequencing primers are described (Supplementary Table S8).

### Statistical analysis

Firstly, *GFAP* mutations were divided into “Neutral” or “Deleterious” using the prediction tool PROVEAN^36^ based on sequence conservation. “Deleterious” means that the mutation is considered selectively deleterious because its alignment-based score among species is equal to or below a predefined threshold. “Neutral” means that the mutation is considered selectively neutral because its alignment-based score is above the threshold. The mean AAO was compared between patients with “Neutral” or “Deleterious” mutations using the *t*-test (R v.3.2.2)^37^.

Next, to identify variants related to AAO of AxD, we performed association analysis with logistic regression using WES and microarray data (Fig. 4 upper). The dependent variable was AAO as categorical data (a younger (<45 years) or older (≥45 years) onset). The independent variables were genotypes of each variant. The covariate was the predicted pathogenicity of *GFAP* mutations as binary categorical data (“Neutral” or “Deleterious”). Association analysis for additive, dominant, and recessive models and QTL analysis for age at onset were performed using PLINK v.1.07 software^38^. The additive model tested the effects of a minor allele dosage (0, 1, 2). Dominant and recessive models tested the effects of a minor allele between (DD, Dd) versus dd, and DD versus (Dd, dd), respectively (D and d are the minor and major alleles, respectively).

Finally, we focused on the candidate genes related to AxD pathophysiology (Fig. 4 lower). The *p*-values of all variations in the candidate genes were extracted from WES and microarray data. A list of candidate genes was created based on a search of PubMed using the keywords “Alexander disease” and “GFAP”. We also hand-searched relevant publications and articles (Supplementary Table S5).

## Supplementary information

supplementary material
