# Supplementary material for: Towards genomic database of Alexander disease to identify variations modifying disease phenotype
Source: Sci Rep. 2019 Oct 14;9:14763. doi: 10.1038/s41598-019-51390-8 (PMC6791890; doi:10.1038/s41598-019-51390-8)
Supplement: Supplementary file 1 — supplementary material [file 41598_2019_51390_MOESM1_ESM.docx]

Title: Towards genomic database of Alexander disease to identify variations modifying disease phenotype

Rei Yasuda,^1^ Masakazu Nakano,^2^ Tomokatsu Yoshida,^1*^ Ryuichi Sato,^2^ Hiroko Adachi,^2^ Yuichi Tokuda,^2^ Ikuko Mizuta,^1^ Kozo Saito,^1^ Jun Matsuura,^1^ Masanori Nakagawa,^3^ Kei Tashiro,^2*^ & Toshiki Mizuno^1^

^1^Department of Neurology, Graduate School of Medical Science, Kyoto Prefectural University of Medicine, Kyoto, Japan

^2^Department of Genomic Medical Sciences, Kyoto Prefectural University of Medicine, Kyoto, Japan

^3^Department of Neurology, North Medical Center, Kyoto Prefectural University of Medicine, Kyoto, Japan

Rei Yasuda and Masakazu Nakano contributed equally to this work.

* Corresponding authors

Table S1. Summary of whole-exome sequencing analysis in association with age at onset.

| Chr | Position | dbSNP ID | Ref/Alt | Minor Allele | MAF | Amino Acid Change | Gene Name | Younger Onset Genotype^a^ | Older Onset Genotype^a^ | *p*-value | Odds Ratio^b^ | Analysis Model |
| --- | --- | --- | --- | --- | --- | --- | --- | --- | --- | --- | --- | --- |
|  |  |  |  |  |  |  |  |  |  |  |  |  |
| 5 | 1081767 | rs4526148 | C/T | C | 0.29 | p.Ala408Thr | *SLC12A7* | 1/1/11 | 3/9/6 | 3.23×10^-3^ | 0.02 | Dominant |
| 10 | 101977883 | rs2230804 | C/T | T | 0.42 | p.Val268Ile | *CHUK* | 1/7/5 | 11/5/2 | 3.45×10^-3^ | 45.00 | Dominant |
| 2 | 85622059 | rs6886 | T/C | T | 0.47 | p.His335Arg | *CAPG* | 5/7/1 | 4/4/10 | 3.63×10^-3^ | 44.56 | Dominant |
| 6 | 138539183 | rs3841283 | A/AG | AG | 0.29 | p.Leu117_Gly118fs | *PBOV1* | 2/11/0 | 13/3/2 | 4.36×10^-3^ | 21.78 | Dominant |
| 6 | 138539315 | rs6927706 | A/G | G | 0.29 | p.Ile73Thr | *PBOV1* | 2/11/0 | 13/3/2 | 4.36×10^-3^ | 21.78 | Dominant |
| 6 | 138539556 | rs11758517 | T/C | C | 0.29 | premature start | *PBOV1* | 2/11/0 | 13/3/2 | 4.36×10^-3^ | 21.78 | Dominant |
| 20 | 47859217 | rs1130146 | G/A | A | 0.35 | p.Gly766Ser | *DDX27* | 2/7/4 | 13/3/2 | 4.36×10^-3^ | 21.78 | Dominant |
| 4 | 110902111 | rs11569017 | A/T | T | 0.29 | p.Asp784Val | *EGF* | 3/7/3 | 13/5/0 | 4.99×10^-3^ | 15.58 | Additive |
| 3 | 33877626 | rs3792594 | G/A | A | 0.32 | p.Ala314Thr | *PDCD6IP* | 2/9/2 | 12/5/1 | 5.08×10^-3^ | 21.12 | Dominant |
| 6 | 138584685 | rs7764091 | T/G | G | 0.31 | p.Ser689Ala | *KIAA1244* | 2/11/0 | 12/4/2 | 5.08×10^-3^ | 21.12 | Dominant |
| 16 | 3808052 | - | TA/T | T | 0.23 | c.3256delT | *CREBBP* | 11/2/0 | 6/12/0 | 5.08×10^-3^ | 0.05 | Dominant |
| 17 | 505129 | rs61644407 | A/C | C | 0.24 | p.Leu375Arg | *VPS53* | 3/9/1 | 15/2/1 | 5.24×10^-3^ | 15.91 | Dominant |
| 3 | 33905566 | rs1127732 | C/T | T | 0.34 | p.Ser735Leu | *PDCD6IP* | 2/9/2 | 11/6/1 | 5.65×10^-3^ | 20.70 | Dominant |
| 16 | 55880480 | rs11860456 | A/C | C | 0.26 | p.Asp537Glu | *CES5A* | 11/1/1 | 7/9/2 | 5.65×10^-3^ | 0.05 | Dominant |
| 22 | 26860269 | rs2014410 | G/C | C | 0.34 | p.Leu443Val | *HPS4* | 2/9/2 | 11/6/1 | 5.65×10^-3^ | 20.70 | Dominant |
| 19 | 58117083 | rs9677004 | A/G | G | 0.26 | p.Thr64Ala | *ZNF530* | 3/9/1 | 14/3/1 | 5.78×10^-3^ | 29.34 | Dominant |
| 19 | 58144715 | rs9749449 | A/G | G | 0.26 | start lost | *ZNF211* | 3/9/1 | 14/3/1 | 5.78×10^-3^ | 29.34 | Dominant |
| 19 | 58151286 | rs4801508 | T/C | C | 0.26 | p.Ile88Thr | *ZNF211* | 3/9/1 | 14/3/1 | 5.78×10^-3^ | 29.34 | Dominant |
| 19 | 58152795 | rs11879465 | C/T | T | 0.26 | p.Ala379Val | *ZNF211* | 3/9/1 | 14/3/1 | 5.78×10^-3^ | 29.34 | Dominant |
| 1 | 158450314 | rs6679056 | A/G | A | 0.42 | p.Glu216Gly | *OR10R2* | 5/6/2 | 2/6/10 | 6.11×10^-3^ | 20.39 | Dominant |
| 1 | 158450382 | rs1418843 | C/T | C | 0.42 | p.Leu239Phe | *OR10R2* | 5/6/2 | 2/6/10 | 6.11×10^-3^ | 20.39 | Dominant |
| 1 | 158549492 | rs863362 | C/T | C | 0.42 | p.Trp66* | *OR10X1* | 5/6/2 | 2/6/10 | 6.11×10^-3^ | 20.39 | Dominant |
| 1 | 158549511 | rs863363 | A/G | A | 0.42 | p.Ile60Thr | *OR10X1* | 5/6/2 | 2/6/10 | 6.11×10^-3^ | 20.39 | Dominant |
| 10 | 73550117 | rs11592462 | C/G | G | 0.18 | p.Thr1999Ser | *CDH23* | 11/2/0 | 9/9/0 | 6.49×10^-3^ | 0.05 | Dominant |
| 2 | 75923413 | rs7560262 | T/C | C | 0.29 | p.Asn80Ser | *GCFC2* | 3/9/1 | 13/3/2 | 6.54×10^-3^ | 28.46 | Dominant |
| 1 | 248309356 | rs73141283 | A/G | G | 0.23 | p.Lys303Glu | *OR2M5* | 12/1/0 | 6/11/1 | 6.62×10^-3^ | 0.04 | Dominant |
| 9 | 79318367 | rs4745571 | T/C | C | 0.21 | p.Asn2721Ser | *PRUNE2* | 4/7/2 | 16/2/0 | 6.83×10^-3^ | 23.98 | Additive |
| 9 | 33240225 | rs706107 | G/A | A | 0.26 | p.Val7Ile | *SPINK4* | 10/3/0 | 6/11/1 | 7.12×10^-3^ | 0.04 | Dominant |
| 2 | 216190020 | rs2372536 | C/G | G | 0.23 | p.Thr116Ser | *ATIC* | 4/7/2 | 15/3/0 | 7.27×10^-3^ | 25.28 | Additive |
| 3 | 69230061 | rs9831516 | G/A | G | 0.31 | p.Ser947Leu | *FRMD4B* | 0/3/10 | 2/12/4 | 7.50×10^-3^ | 0.07 | Dominant |
| 15 | 40659476 | rs35043215 | G/A | A | 0.21 | p.Gly388Glu | *DISP2* | 12/1/0 | 7/10/1 | 7.52×10^-3^ | 0.04 | Dominant |
| 6 | 32731258 | rs1573649 | G/A | G | 0.32 | premature start | *HLA-DQB2* | 2/8/3 | 1/6/11 | 7.58×10^-3^ | 27.51 | Dominant |
| 10 | 69934258 | rs3814182 | C/G | G | 0.27 | p.Ser803Arg | *MYPN* | 10/3/0 | 7/8/3 | 7.58×10^-3^ | 0.04 | Dominant |
| 4 | 2744087 | rs2269495 | G/A | A | 0.24 | p.Ala289Val | *TNIP2* | 4/8/1 | 13/5/0 | 7.93×10^-3^ | 12.73 | Additive |
| 3 | 128755814 | rs3732429 | T/C | T | 0.42 | c.1450T>C | *EFCC1* | 2/10/1 | 4/4/10 | 8.21×10^-3^ | 26.57 | Dominant |
| 22 | 30953295 | rs2267161 | C/T | T | 0.39 | p.Val29Met | *GAL3ST1* | 1/10/2 | 10/6/2 | 8.21×10^-3^ | 26.57 | Dominant |
| 1 | 16577908 | rs12069239 | G/C | C | 0.16 | p.Pro471Ala | *FBXO42* | 12/1/0 | 9/9/0 | 8.75×10^-3^ | 0.04 | Dominant |
| 1 | 40980731 | rs11208299 | G/T | G | 0.42 | p.Gly172Val | *EXO5* | 2/10/1 | 3/6/9 | 8.75×10^-3^ | 26.18 | Dominant |
| 4 | 110667590 | rs74817407 | C/T | T | 0.16 | p.Arg406His | *CFI* | 12/1/0 | 9/9/0 | 8.75×10^-3^ | 0.04 | Dominant |
| 15 | 101110096 | rs12915007 | T/C | C | 0.26 | p.Ile541Val | *LINS* | 4/6/3 | 14/4/0 | 9.17×10^-3^ | 10.80 | Additive |
| 17 | 7673605 | rs11868946 | A/G | G | 0.27 | p.Glu1326Gly | *DNAH2* | 4/7/2 | 12/6/0 | 9.40×10^-3^ | 12.06 | Additive |
| 4 | 110883121 | rs11568943 | G/A | A | 0.26 | p.Arg431Lys | *EGF* | 3/9/1 | 13/5/0 | 9.59×10^-3^ | 13.45 | Dominant |
| 9 | 130191186 | rs13292096 | C/T | T | 0.34 | p.Thr7Ile | *ZNF79* | 10/1/2 | 5/10/3 | 9.59×10^-3^ | 0.07 | Dominant |
| 9 | 130197414 | rs4504745 | A/G | G | 0.34 | p.Arg27Gly | *ZNF79* | 10/1/2 | 5/10/3 | 9.59×10^-3^ | 0.07 | Dominant |
| 13 | 77574983 | rs1800209 | A/G | G | 0.29 | p.Lys368Arg | *CLN5* | 10/3/0 | 5/11/2 | 9.59×10^-3^ | 0.07 | Dominant |
| 11 | 64109118 | rs647152 | T/G | G | 0.37 | p.Asp193Glu | *CCDC88B* | 9/2/2 | 3/13/2 | 9.60×10^-3^ | 0.05 | Dominant |

The variants with a *p*-value <0.01 in either additive, dominant, or recessive models are shown.

^a^ The number of patients with reference allele homozygous / heterozygous / alternative allele homozygous is shown.

^b^ Odds ratio represents the odds of a younger onset with the minor compared with major allele.

Chr=chromosome; Ref=reference allele; Alt=alternative allele; MAF=minor allele frequency.

Table S2. Summary of microarray analysis in association with age at onset.

| Chr | Position | dbSNP ID | Minor/Major allele | MAF | Gene Name | Younger Onset Genotype^a^ | Older Onset Genotype^a^ | *p*-value | Odds Ratio^b^ | Analysis Model |
| --- | --- | --- | --- | --- | --- | --- | --- | --- | --- | --- |
|  |  |  |  |  |  |  |  |  |  |  |
| 3 | 110699266 | rs2169564 | T/G | 0.32 |  | 0/1/12 | 2/15/1 | 5.88×10^-4^ | 0.01 | Dominant |
| 4 | 176660420 | rs13152426 | A/G | 0.31 | *GPM6A* | 0/2/11 | 1/15/2 | 1.41×10^-3^ | 0.03 | Dominant |
| 9 | 9785685 | rs2382056 | G/A | 0.24 | *PTPRD* | 1/10/2 | 1/1/16 | 1.41×10^-3^ | 33.07 | Dominant |
| 9 | 9787587 | rs10977976 | A/G | 0.23 | *PTPRD* | 0/11/2 | 1/1/16 | 1.41×10^-3^ | 33.07 | Dominant |
| 6 | 138929434 | rs9484186 | T/C | 0.24 |  | 1/10/2 | 0/3/15 | 1.46×10^-3^ | 61.50 | Additive |
| 12 | 52657665 | rs1902763 | A/G | 0.27 |  | 0/1/12 | 3/10/5 | 1.75×10^-3^ | 0.01 | Dominant |
| 19 | 40272816 | rs10518261 | G/A | 0.24 | *LEUTX* | 0/1/12 | 1/12/5 | 1.75×10^-3^ | 0.01 | Dominant |
| 8 | 53488242 | rs7826532 | A/G | 0.23 |  | 0/1/12 | 1/11/6 | 1.83×10^-3^ | 0.01 | Dominant |
| 5 | 89261270 | rs10514312 | A/G | 0.26 |  | 2/9/2 | 0/3/15 | 2.34×10^-3^ | 25.31 | Dominant |
| 21 | 46292384 | rs2248034 | C/T | 0.24 | *PTTG1IP* | 0/11/2 | 1/2/15 | 2.34×10^-3^ | 25.31 | Dominant |
| 16 | 27832537 | rs1363750 | T/G | 0.27 | *GSG1L* | 1/11/1 | 0/4/14 | 2.42×10^-3^ | 48.55 | Dominant |
| 16 | 80456403 | rs10514496 | C/T | 0.40 |  | 0/3/10 | 5/12/1 | 2.42×10^-3^ | 0.02 | Dominant |
| 4 | 130606797 | rs4864109 | G/A | 0.31 |  | 0/2/11 | 3/11/4 | 2.72×10^-3^ | 0.02 | Dominant |
| 3 | 179424410 | rs17788187 | T/G | 0.26 | *USP13* | 1/11/1 | 0/3/15 | 2.75×10^-3^ | 46.59 | Dominant |
| 2 | 49498006 | rs9636436 | A/G | 0.32 |  | 3/9/1 | 0/5/13 | 2.90×10^-3^ | 46.67 | Dominant |
| 3 | 104057483 | rs7620614 | A/G | 0.26 |  | 0/1/12 | 2/11/5 | 2.90×10^-3^ | 0.02 | Dominant |
| 9 | 124125520 | rs17086 | G/A | 0.31 | *STOM* | 1/0/12 | 4/9/5 | 2.90×10^-3^ | 0.02 | Dominant |
| 19 | 39447119 | rs10412049 | T/C | 0.26 | *FBXO17* | 0/1/12 | 2/11/5 | 2.90×10^-3^ | 0.02 | Dominant |
| 3 | 73425846 | rs6777921 | C/A | 0.29 |  | 2/9/2 | 0/5/13 | 3.02×10^-3^ | 46.64 | Dominant |
| 8 | 73415017 | rs1554781 | A/G | 0.27 |  | 1/10/2 | 0/5/13 | 3.02×10^-3^ | 46.64 | Dominant |
| 2 | 106358929 | rs4851837 | T/G | 0.24 |  | 0/1/12 | 2/10/6 | 3.22×10^-3^ | 0.02 | Dominant |
| 9 | 86368105 | rs10868053 | A/G | 0.35 | *GKAP1* | 3/9/1 | 1/5/12 | 3.22×10^-3^ | 45.64 | Dominant |
| 9 | 86399815 | rs7021357 | C/T | 0.35 | *GKAP1* | 3/9/1 | 1/5/12 | 3.22×10^-3^ | 45.64 | Dominant |
| 5 | 1081767 | - | C/T | 0.29 | *SLC12A7* | 1/1/11 | 3/9/6 | 3.23×10^-3^ | 0.02 | Dominant |
| 12 | 32424230 | rs11051891 | G/A | 0.32 | *BICD1* | 0/11/2 | 3/3/12 | 3.23×10^-3^ | 46.00 | Dominant |
| 13 | 77104061 | rs1146901 | C/T | 0.29 |  | 1/1/11 | 3/9/6 | 3.23×10^-3^ | 0.02 | Dominant |
| 10 | 10240975 | rs1570411 | T/C | 0.19 |  | 0/10/3 | 0/2/16 | 3.39×10^-3^ | 20.25 | Dominant |
| 18 | 48177860 | rs11662176 | T/C | 0.26 | *MAPK4* | 3/7/3 | 1/1/16 | 3.39×10^-3^ | 20.25 | Dominant |
| 2 | 31166795 | rs10179187 | G/A | 0.24 | *GALNT14* | 3/7/3 | 0/2/16 | 3.39×10^-3^ | 34.43 | Dominant |
| 2 | 34974141 | rs11683299 | C/T | 0.23 |  | 0/10/3 | 2/0/16 | 3.39×10^-3^ | 34.43 | Dominant |
| 4 | 13914373 | rs10805321 | A/C | 0.21 |  | 1/9/3 | 0/2/16 | 3.39×10^-3^ | 34.43 | Dominant |
| 6 | 66957869 | rs9453647 | T/G | 0.45 |  | 2/1/10 | 7/9/2 | 3.39×10^-3^ | 0.03 | Dominant |
| 9 | 35191713 | rs3904435 | G/A | 0.32 | *UNC13B* | 0/3/10 | 1/15/2 | 3.39×10^-3^ | 0.03 | Dominant |
| 20 | 6842330 | rs7268985 | C/T | 0.34 |  | 0/3/10 | 2/14/2 | 3.39×10^-3^ | 0.03 | Dominant |
| 8 | 138495720 | rs6577800 | G/A | 0.23 |  | 0/2/11 | 1/10/7 | 3.39×10^-3^ | 0.02 | Dominant |
| 4 | 185520068 | rs3970313 | G/A | 0.32 |  | 0/2/11 | 4/10/4 | 3.44×10^-3^ | 0.04 | Dominant |
| 5 | 165107100 | rs516732 | T/C | 0.31 |  | 1/1/11 | 2/12/4 | 3.44×10^-3^ | 0.04 | Dominant |
| 7 | 52582500 | rs10280700 | G/A | 0.31 |  | 3/8/2 | 1/3/14 | 3.44×10^-3^ | 22.91 | Dominant |
| 7 | 114203949 | rs12532920 | G/A | 0.27 | *FOXP2* | 1/10/2 | 1/3/14 | 3.44×10^-3^ | 22.91 | Dominant |
| 7 | 114211447 | rs10269986 | A/G | 0.27 | *FOXP2* | 1/10/2 | 1/3/14 | 3.44×10^-3^ | 22.91 | Dominant |
| 7 | 114254407 | rs10230087 | G/A | 0.27 | *FOXP2* | 1/10/2 | 1/3/14 | 3.44×10^-3^ | 22.91 | Dominant |
| 12 | 53323694 | rs2638490 | G/A | 0.26 | *KRT8* | 0/2/11 | 0/14/4 | 3.44×10^-3^ | 0.04 | Dominant |
| 20 | 6860895 | rs1028394 | T/C | 0.27 |  | 0/2/11 | 1/13/4 | 3.44×10^-3^ | 0.04 | Dominant |
| 5 | 89260057 | rs6861634 | C/T | 0.39 |  | 3/9/1 | 2/5/11 | 3.45×10^-3^ | 45.00 | Dominant |
| 8 | 14640051 | rs2253225 | C/T | 0.37 | *SGCZ* | 1/11/1 | 3/4/11 | 3.45×10^-3^ | 45.00 | Dominant |
| 10 | 101911142 | rs12806 | G/A | 0.40 | *ERLIN1* | 4/8/1 | 2/5/11 | 3.45×10^-3^ | 45.00 | Dominant |
| 10 | 101977883 | - | T/C | 0.42 | *CHUK* | 5/7/1 | 2/5/11 | 3.45×10^-3^ | 45.00 | Dominant |
| 10 | 102016268 | rs2270961 | T/C | 0.42 | *CWF19L1* | 5/7/1 | 2/5/11 | 3.45×10^-3^ | 45.00 | Dominant |
| 6 | 165295240 | rs9347937 | C/T | 0.34 |  | 1/10/2 | 1/7/10 | 3.52×10^-3^ | 45.24 | Dominant |
| 17 | 26134974 | rs12709500 | C/T | 0.39 |  | 2/9/2 | 3/5/10 | 3.52×10^-3^ | 45.24 | Dominant |

Variants with the top 50 *p*-values in either additive, dominant, or recessive models are shown.

^a^ The number of the patients with minor allele homozygous / heterozygous / major allele homozygous is shown.

^b^ Odds ratio represents the odds of a younger onset with the minor allele compared with major allele.

Chr=chromosome; MAF=minor allele frequency.

Supplementary materials S3.

As clinical symptoms of the bulbospinal type AxD are comparatively mild, the mutations that cause this type are considered to have less severe effects. If these mutations are not so harmful, they could be affected by genetic modifiers. Therefore, we searched for modifiers only in 19 bulbospinal type patients.

Whole exome sequencing analysis of the quantitative trait locus (QTL) for age at onset revealed 16 variants with a p-value <1.0×10^-5^ (Table S3-1) and microarray analysis revealed 22 variants with a p-value <1.0×10^-5^ (Table S3-2). However, as shown in Figs. S3-1 and S3-2, unique variants of the patient with an extremely early age at onset (Patient 13, E362G) markedly affected the results. Subsequently, we excluded the outlier and performed the same analysis (Supplementary materials S4).

Table S3-1. Summary of whole exome sequencing analysis of quantitative trait locus for age at onset in 19 bulbospinal type AxD patients.

| Chr | Position | Ref/Alt | Minor Allele | MAF | Amino Acid Change | Gene Name | Genotype^a^ | R2 | *p*-value |
| --- | --- | --- | --- | --- | --- | --- | --- | --- | --- |
| 1 | 48459907 | C/T | T | 0.13 | p.Ala155Ala | *TRABD2B* | 15/3/1 | 0.80 | 2.05×10^-7^ |
| 1 | 230895340 | C/A | A | 0.08 | p.Ser122Arg | *CAPN9* | 17/1/1 | 0.69 | 9.37×10^-6^ |
| 3 | 126741011 | G/A | A | 0.11 | p.Thr1374Thr | *PLXNA1* | 16/2/1 | 0.75 | 1.76×10^-6^ |
| 7 | 127347733 | G/C | C | 0.08 | c.1038+32G>C | *SND1* | 17/1/1 | 0.69 | 9.37×10^-6^ |
| 7 | 127361269 | G/A | A | 0.08 | c.1039-72G>A | *SND1* | 17/1/1 | 0.69 | 9.37×10^-6^ |
| 7 | 127447499 | A/T | A | 0.08 | c.1153-39A>T | *SND1* | 1/1/17 | 0.69 | 9.37×10^-6^ |
| 7 | 127669584 | T/C | C | 0.08 | p.Ala370Ala | *LRRC4* | 17/1/1 | 0.69 | 9.37×10^-6^ |
| 10 | 95095600 | G/C | G | 0.08 | c.4563+78C>G | *MYOF* | 1/1/17 | 0.69 | 9.37×10^-6^ |
| 10 | 95095667 | A/G | A | 0.08 | c.4563+11T>C | *MYOF* | 1/1/17 | 0.69 | 9.37×10^-6^ |
| 10 | 95097537 | T/C | T | 0.08 | c.4437+35A>G | *MYOF* | 1/1/17 | 0.69 | 9.37×10^-6^ |
| 10 | 95126406 | A/G | G | 0.08 | c.2590-94T>C | *MYOF* | 17/1/1 | 0.69 | 9.37×10^-6^ |
| 12 | 9010671 | G/A | A | 0.08 | p.Val588Val | *A2ML1* | 17/1/1 | 0.69 | 9.37×10^-6^ |
| 13 | 32706028 | T/C | C | 0.08 | c.885+51T>C | *FRY* | 17/1/1 | 0.69 | 9.37×10^-6^ |
| 16 | 20328685 | T/C | C | 0.08 | p.Gln425Gln | *GP2* | 17/1/1 | 0.69 | 9.37×10^-6^ |
| 22 | 24342338 | C/G | G | 0.08 | n.313G>C | *GSTTP1* | 17/1/1 | 0.69 | 9.37×10^-6^ |
| 22 | 37678565 | G/A | A | 0.26 | c.-46G>A | *CYTH4* | 10/8/1 | 0.71 | 6.18×10^-6^ |

The variants with a *p*-value <1.0×10^-5^ are shown.

^a^ The number of patients with reference allele homozygous / heterozygous / alternative allele homozygous is shown.

Chr=chromosome; Ref=reference allele; Alt=alternative allele; MAF=minor allele frequency.


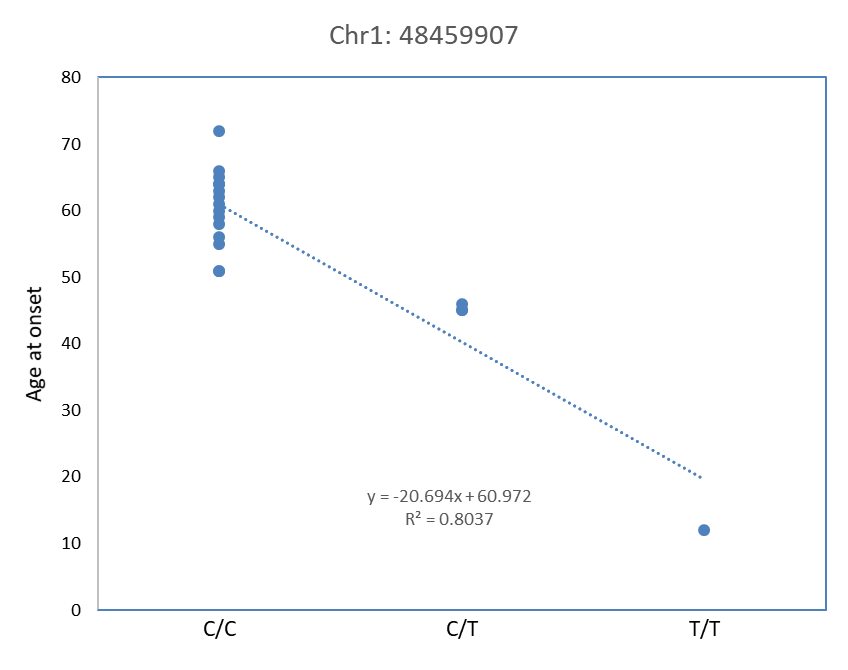


Fig S3-1. Relationship between the genotype of the SNP and age at onset in whole exome sequencing analysis of QTL for age at onset in 19 bulbospinal type AxD patients.

Table S3-2. Summary of microarray analysis of quantitative trait locus for age at onset in 19 bulbospinal type AxD patients.

| Chr | Position | SNP ID | Minor/Major Allele | MAF | Gene Name | Genotype^a^ | R2 | *p*-value |
| --- | --- | --- | --- | --- | --- | --- | --- | --- |
| 1 | 48480638 | rs12137714 | C/T | 0.16 |  | 1/4/14 | 0.72 | 4.81×10^-6^ |
| 1 | 230895340 | exm158172 | A/C | 0.08 | *CAPN9* | 1/1/17 | 0.69 | 9.37×10^-6^ |
| 3 | 25944830 | rs4334607 | G/A | 0.08 |  | 1/1/17 | 0.69 | 9.37×10^-6^ |
| 5 | 4243226 | rs17723822 | G/T | 0.08 |  | 1/1/17 | 0.69 | 9.37×10^-6^ |
| 5 | 17118930 | rs1032763 | T/C | 0.13 |  | 1/3/15 | 0.72 | 3.91×10^-6^ |
| 7 | 46753491 | rs700750 | G/T | 0.08 |  | 1/1/17 | 0.69 | 9.37×10^-6^ |
| 7 | 46778653 | rs957755 | A/C | 0.08 |  | 1/1/17 | 0.69 | 9.37×10^-6^ |
| 7 | 98273346 | rs879187 | T/C | 0.11 |  | 1/2/16 | 0.73 | 2.80×10^-6^ |
| 7 | 98304639 | rs6950545 | C/T | 0.13 |  | 1/3/15 | 0.72 | 3.91×10^-6^ |
| 7 | 98312921 | rs10238278 | G/A | 0.11 |  | 1/2/16 | 0.73 | 2.80×10^-6^ |
| 7 | 127339415 | rs741599 | C/A | 0.08 | *SND1* | 1/1/17 | 0.69 | 9.37×10^-6^ |
| 7 | 127464386 | rs325446 | G/A | 0.08 | *SND1* | 1/1/17 | 0.69 | 9.37×10^-6^ |
| 7 | 127607474 | rs720300 | C/T | 0.08 | *SND1* | 1/1/17 | 0.69 | 9.37×10^-6^ |
| 10 | 95102221 | rs787695 | A/G | 0.08 | *MYOF* | 1/1/17 | 0.69 | 9.37×10^-6^ |
| 10 | 95124673 | rs787640 | G/A | 0.08 | *MYOF* | 1/1/17 | 0.69 | 9.37×10^-6^ |
| 10 | 95134850 | rs2298156 | G/T | 0.08 | *MYOF* | 1/1/17 | 0.69 | 9.37×10^-6^ |
| 10 | 95134959 | rs2298155 | G/A | 0.08 | *MYOF* | 1/1/17 | 0.69 | 9.37×10^-6^ |
| 10 | 95139526 | rs10882229 | A/G | 0.11 | *MYOF* | 1/2/16 | 0.75 | 1.76×10^-6^ |
| 10 | 95148470 | rs696178 | T/G | 0.08 | *MYOF* | 1/1/17 | 0.69 | 9.37×10^-6^ |
| 12 | 9010671 | exm2251245 | A/G | 0.08 | *A2ML1* | 1/1/17 | 0.69 | 9.37×10^-6^ |
| 13 | 32571088 | rs439716 | A/C | 0.11 |  | 1/2/16 | 0.75 | 1.76×10^-6^ |
| 22 | 37678565 | rs3213557 | A/G | 0.26 | *CYTH4* | 1/8/10 | 0.71 | 6.18×10^-6^ |

The variants with a *p*-value <1.0×10^-5^ are shown.

^a^ The number of the patients with minor allele homozygous / heterozygous / major allele homozygous is shown.

Chr=chromosome; MAF=minor allele frequency.


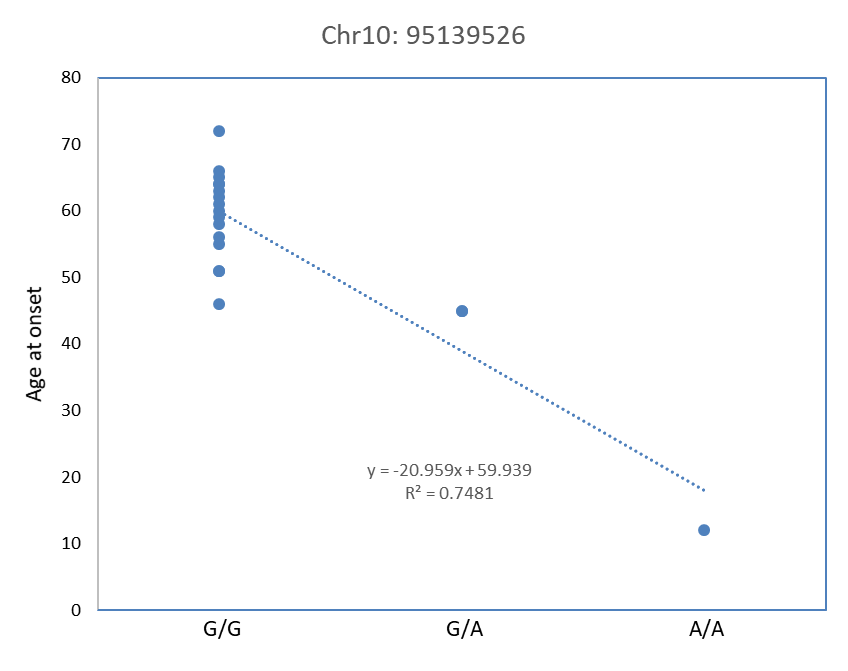


Fig S3-2. Relationship between the genotype of the SNP and age at onset in microarray analysis of QTL for age at onset in 19 bulbospinal type AxD patients.

Supplementary materials S4.

The search for modifier genes in bulbospinal type patients was markedly affected by unique variants of the patient with an extremely early age at onset (Supplementary materials S3). Therefore, we excluded the outlier (Patient 13, E362G) and searched for modifiers in 18 bulbospinal type patients.

Whole exome sequencing analysis of quantitative trait locus (QTL) for age at onset revealed 14 variants with p-value <1.0×10^-4^ (Table S4-1) and microarray analysis revealed 42 variants with p-value <1.0×10^-4^ (Table S4-2).

Table S4-1. Summary of whole exome sequencing analysis of quantitative trait locus for age at onset in 18 bulbospinal type AxD patients after excluding the outlier.

| Chr | Position | Ref/Alt | Minor Allele | MAF | Amino Acid Change | Gene Name | Genotype^a^ | R2 | *p*-value |
| --- | --- | --- | --- | --- | --- | --- | --- | --- | --- |
| 3 | 75786387 | T/C | C | 0.17 | p.Tyr796Cys | *ZNF717* | 12/6/0 | 0.65 | 5.51×10^-5^ |
| 3 | 75786403 | A/T | T | 0.17 | p.Cys791Ser | *ZNF717* | 12/6/0 | 0.65 | 5.51×10^-5^ |
| 3 | 113955820 | G/C | G | 0.47 | p.Ser34Ser | *ZNF80* | 4/9/5 | 0.64 | 6.24×10^-5^ |
| 6 | 24437524 | C/T | T | 0.44 | c.2021G>A | *GPLD1* | 6/8/4 | 0.67 | 3.58×10^-5^ |
| 6 | 24445829 | G/A | A | 0.44 | p.His655His | *GPLD1* | 6/8/4 | 0.67 | 3.58×10^-5^ |
| 6 | 24454196 | G/T | T | 0.44 | c.1335+47C>A | *GPLD1* | 6/8/4 | 0.67 | 3.58×10^-5^ |
| 6 | 24475517 | TATTA/T | T | 0.28 | c.331-62_331-59delTAAT | *GPLD1* | 10/6/2 | 0.66 | 4.73×10^-5^ |
| 6 | 24828490 | C/T | T | 0.17 | p.Arg847Gln | *FAM65B* | 12/6/0 | 0.62 | 9.68×10^-5^ |
| 9 | 113550172 | A/G | G | 0.11 | c.1669+54A>G | *MUSK* | 14/4/0 | 0.63 | 9.14×10^-5^ |
| 9 | 113562649 | A/G | G | 0.11 | p.Asn578Ser | *MUSK* | 14/4/0 | 0.63 | 9.14×10^-5^ |
| 11 | 3712537 | T/C | C | 0.11 | c.4454+40A>G | *NUP98* | 14/4/0 | 0.63 | 9.14×10^-5^ |
| 17 | 20363552 | C/A | A | 0.11 | c.131+113G>T | *LGALS9B* | 14/4/0 | 0.63 | 9.14×10^-5^ |
| 20 | 41306600 | A/G | G | 0.39 | p.Asp353Asp | *PTPRT* | 7/8/3 | 0.65 | 5.96×10^-5^ |
| 21 | 11114805 | C/G | G | 0.14 |  |  | 13/5/0 | 0.72 | 8.57×10^-6^ |

The variants with a *p*-value <1.0×10^-4^ are shown.

^a^ The number of patients with reference allele homozygous / heterozygous / alternative allele homozygous is shown.

Chr=chromosome; Ref=reference allele; Alt=alternative allele; MAF=minor allele frequency.

Table S4-2. Summary of microarray analysis of the quantitative trait locus for age at onset in 18 bulbospinal type AxD patients after excluding the outlier.

| Chr | Position | SNP ID | Minor/Major allele | MAF | Gene Name | Genotype | R2 | *p*-value |
| --- | --- | --- | --- | --- | --- | --- | --- | --- |
| 1 | 17486722 | rs4620585 | C/T | 0.4167 |  | 3/9/6 | 0.63 | 8.63×10^-5^ |
| 1 | 34982041 | rs6694043 | A/G | 0.1944 |  | 0/7/11 | 0.65 | 4.97×10^-5^ |
| 1 | 54575746 | rs4927055 | T/C | 0.4722 |  | 3/11/4 | 0.63 | 8.97×10^-5^ |
| 1 | 57246662 | rs1774822 | C/T | 0.4444 | *C1orf168* | 4/8/6 | 0.65 | 4.86×10^-5^ |
| 1 | 108356000 | rs17485868 | A/G | 0.4722 | *VAV3* | 5/7/6 | 0.63 | 8.93×10^-5^ |
| 1 | 193772404 | rs1410394 | C/T | 0.1111 |  | 0/4/14 | 0.63 | 9.14×10^-5^ |
| 1 | 193792484 | exm2268962 | T/C | 0.1111 |  | 0/4/14 | 0.63 | 9.14×10^-5^ |
| 1 | 193792484 | rs1575938 | T/C | 0.1111 |  | 0/4/14 | 0.63 | 9.14×10^-5^ |
| 1 | 193895333 | rs4330903 | T/C | 0.1111 |  | 0/4/14 | 0.63 | 9.14×10^-5^ |
| 1 | 193901655 | rs4657892 | G/A | 0.1111 |  | 0/4/14 | 0.63 | 9.14×10^-5^ |
| 1 | 193917413 | rs6680840 | C/T | 0.1111 |  | 0/4/14 | 0.63 | 9.14×10^-5^ |
| 1 | 193923694 | rs1570442 | A/G | 0.1111 |  | 0/4/14 | 0.63 | 9.14×10^-5^ |
| 1 | 227586401 | rs6659701 | A/G | 0.5 |  | 3/12/3 | 0.62 | 9.92×10^-5^ |
| 2 | 36082275 | rs940386 | A/C | 0.1667 |  | 0/6/12 | 0.73 | 7.16×10^-6^ |
| 2 | 221110780 | rs1993313 | T/G | 0.3889 |  | 3/8/7 | 0.64 | 7.35×10^-5^ |
| 2 | 230935754 | rs12475755 | A/G | 0.1111 |  | 0/4/14 | 0.63 | 9.14×10^-5^ |
| 3 | 2796414 | rs3897737 | C/T | 0.4444 | *CNTN4* | 3/10/5 | 0.63 | 8.83×10^-5^ |
| 3 | 113958599 | rs905569 | C/T | 0.4722 |  | 4/9/5 | 0.64 | 6.24×10^-5^ |
| 3 | 137447485 | exm-rs10935268 | A/G | 0.1111 |  | 0/4/14 | 0.63 | 9.14×10^-5^ |
| 3 | 137447485 | rs10935268 | A/G | 0.1111 |  | 0/4/14 | 0.63 | 9.14×10^-5^ |
| 3 | 176261513 | rs1377820 | A/G | 0.5 |  | 3/12/3 | 0.71 | 1.29×10^-5^ |
| 4 | 25576330 | rs13147366 | T/C | 0.2222 |  | 0/8/10 | 0.62 | 9.50×10^-5^ |
| 4 | 64360700 | rs11131464 | C/T | 0.2222 |  | 2/4/12 | 0.62 | 9.91×10^-5^ |
| 4 | 156038542 | rs12650515 | C/A | 0.3056 |  | 2/7/9 | 0.69 | 2.02×10^-5^ |
| 6 | 24445829 | kgp10749727 | A/G | 0.4444 | *GPLD1* | 4/8/6 | 0.67 | 3.58×10^-5^ |
| 6 | 24454196 | rs9393558 | T/G | 0.4444 | *GPLD1* | 4/8/6 | 0.67 | 3.58×10^-5^ |
| 6 | 24828490 | exm520450 | T/C | 0.1667 | *FAM65B* | 0/6/12 | 0.62 | 9.68×10^-5^ |
| 6 | 24828490 | rs12183109 | T/C | 0.1667 | *FAM65B* | 0/6/12 | 0.62 | 9.68×10^-5^ |
| 6 | 50357617 | rs1994785 | C/T | 0.1944 |  | 0/7/11 | 0.68 | 2.72×10^-5^ |
| 6 | 109075953 | rs1268160 | T/C | 0.1111 | *LINC00222* | 0/4/14 | 0.63 | 9.14×10^-5^ |
| 6 | 124098943 | exm2262035 | C/A | 0.25 |  | 1/7/10 | 0.72 | 8.03×10^-6^ |
| 8 | 3594906 | rs2623702 | C/T | 0.1111 | *CSMD1* | 0/4/14 | 0.63 | 9.14×10^-5^ |
| 8 | 4012166 | rs2407307 | A/G | 0.1667 | *CSMD1* | 0/6/12 | 0.65 | 5.51×10^-5^ |
| 8 | 21199381 | rs2125649 | G/A | 0.1111 |  | 0/4/14 | 0.63 | 9.14×10^-5^ |
| 8 | 99364717 | exm2270899 | T/C | 0.4444 |  | 3/10/5 | 0.65 | 5.89×10^-5^ |
| 9 | 88756246 | rs11141262 | C/T | 0.25 |  | 1/7/10 | 0.64 | 6.50×10^-5^ |
| 9 | 113562649 | exm771900 | G/A | 0.1111 | *MUSK* | 0/4/14 | 0.63 | 9.14×10^-5^ |
| 16 | 84463189 | rs8059665 | T/G | 0.3333 | *ATP2C2* | 3/6/9 | 0.64 | 6.29×10^-5^ |
| 16 | 84465253 | rs4782962 | T/C | 0.3333 | *ATP2C2* | 3/6/9 | 0.64 | 6.29×10^-5^ |
| 20 | 41281104 | rs6030395 | T/C | 0.3889 | *PTPRT* | 3/8/7 | 0.71 | 1.04×10^-5^ |
| 20 | 41286043 | rs2206426 | A/G | 0.3889 | *PTPRT* | 2/10/6 | 0.65 | 5.79×10^-5^ |
| 20 | 41306600 | rs2425516 | G/A | 0.3889 | *PTPRT* | 3/8/7 | 0.65 | 5.96×10^-5^ |

The variants with a *p*-value <1.0×10^-4^ are shown.

^a^ The number of the patients with minor allele homozygous / heterozygous / major allele homozygous is shown.

Chr=chromosome; MAF=minor allele frequency.

Table S5. Candidate genes associated with the pathophysiology of Alexander disease.

| Gene Symbol | Summary |
| --- | --- |
| *VIM* | Included in RF. ^1^ |
| *NES* | Included in RF. ^1^ |
| *PLEC* | A second binding partner of GFAP and included in RF. ^1^ Insufficient amounts of plectin, due to mutant GFAP expression, promote GFAP aggregation and RF formation in AxD. ^2^ |
| *CRYAB* | A binding partner of GFAP and included in RF. ^1^ Deficiency of αB-crystallin leads to increased mortality and genetically enhanced expression ofαB-crystallin resucued lethal phenotype in the mouse models of AxD. ^3^ |
| *HSP27 (HSPB1, HSPB2)* | Included in RF. ^1^ |
| *p62 (SQSTM1)* | Included in RF. ^1^ |
| *GLT-1 (SLC1A2)* | The major glutamate transporter of astrocytes. Reduction in glutamate transport would render neurons more susceptible to excitotoxic death. ^1,4^ Protein and mRNA reductions for Kir4.1 and GLT-1 are exacerbated in AxD models that demonstrate earlier accumulation of GFAP and increased RF formation. ^5^ In hippocampal astrocytes of three mouse models of AxD, astrocytes activate the mTOR cascade, acquire CD44, and lose GLT-1. ^6^ |
| *NRF2 (NFE2L2)* | Regulates stress response pathway. Known to be activated in both human AxD and mouse models. ^1,7^ Forcing overexpression of Nrf2 in astrocytes of R236H GFAP mutant mice decreased GFAP protein in all brain regions. ^8^ |
| *MLC1* | Causative gene for MLC, an other disorder of astrocytes sharing some neuropathological features with AxD. ^1^ |
| *GLIALCAM (HEPACAM)* | Causative gene for MLC, an other disorder of astrocytes sharing some neuropathological features with AxD. ^1^ |
| *NG2 (CSPG4)* | AxD-related mutations may cause coexpression of GFAP and NG2 in neurosphere cultures, which would inhibit the differentiation of precursors into oligodendrocytes. ^9^ |
| *GAN* | An important factor that targets GFAP for degradation through the proteasome pathway. ^10^ |
| *KCNJ10 (KIR4.1)* | Protein and mRNA reductions for Kir4.1 and GLT-1 are exacerbated in AxD models that demonstrate earlier accumulation of GFAP and increased RF formation. ^5^ |
| *CXCL10* | Cytokine and chemokine analysis showed CXCL10 and CCL2 to be the most and earliest increased molecules in a mouse model of AxD. ^11^ |
| *CCL2* | Cytokine and chemokine analysis showed CXCL10 and CCL2 to be the most and earliest increased molecules in a mouse model of AxD. ^11^ |
| *CUL4B* | A scaffold protein that assembles E3 ubiquitin ligase, represses the expression of GFAP in NPCs during brain development. ^12^ |
| *PTGDS* | The increased generation of GFAP-positive cells from Cul4b-null NPCs was mediated by an upregulation of prostaglandin D2 synthase PTGDS. ^12^ |
| *HDACs* | HDAC inhibition results in changes in transcription, splicing and organization of GFAP. ^13^ |
| *TARDBP* | TDP-43 is present in RFs in AxD patient brains, and insoluble phosphorylated full-length and high molecular weight TDP-43 accumulates in white matter of such brains. ^14^ |
| *CASP6* | GFAP is cleaved specifically by caspase 6 at L12 linker domain in vitro. The N-terminal GFAP forms filamentous structures that are variable in width and prone to aggregation. ^15^ |
| *PTP1B (PTPN1)* | Pharmacological inhibition of the protein-tyrosine phosphatase PTP1B increased keratin 8 Tyr-267 phosphorylation, decreased solubility, and increased K8 filament bundling. The mutation of paralogous Tyr in glial fibrillary acidic protein (GFAP), which is mutated in AxD (Y242D), exhibited highly irregular filament organization and diminished solubility. ^16^ |
| *AP-1 (JUN, FOS)* | The two alleles of polymorphic locus upstream of the *GFAP* transcriptional start site have shown to bind the AP-1 complex to different extents, thus promoting variable transcriptional activities of the GFAP promoter. ^17,18^ |
| *HDAC6* | Among two half-siblings suspected of AxD with a mutation in GFAP-epsilon, one patient with severe motor-neuron disease had a mutation in HDAC6, a candidate motor-neuron disease susceptibility gene. ^19^ |
| *MTOR* | In hippocampal astrocytes of three mouse models of AxD, astrocytes activate the mTOR cascade, acquire CD44, and lose GLT-1. ^6^ |
| *CD44* | In hippocampal astrocytes of three mouse models of AxD, astrocytes activate the mTOR cascade, acquire CD44, and lose GLT-1. ^6^ |
| *SYNM* | Many of the GFAP-positive reactive astrocytes were positive for intermediate filament protein synemin, and synemin was also present in RF. ^20^ |
| *JNK (MAPK8, SAPK)* | Retinoic acid activates specifically the JNK phosphorylation pathway, which in turn inhibits GFAP expression. ^21^ GFAP accumulation led to a decrease of proteasome activity and an activation of the MLK2-JNK pathway. ^22^ |
| *PI3K (PIK3)* | PI3K is upstream to all the key events leading to the expression of GFAP. ^23^ |
| *CASP3* | Some mutations in the C-terminus of GFAP correlate with caspase 3 cleavage and the loss of cell viability, suggesting that these could be contributory factors in the development of AxD. ^24^ |
| *p38 kinase (MAPK14)* | The C-terminal GFAP tail mutants correlates with the activation of the p38 stress-activated protein kinase. ^24^ |
| *MLK2 (MAP3K10)* | GFAP accumulation led to a decrease of proteasome activity and an activation of the MLK2-JNK pathway. ^22^ |
| *PSEN1, PSEN2* | A splice variant of GFAP, GFAP epsilon, interacts with the presenilin proteins. ^25^ |
| *EDN1* | Endothelin-1 induces astrocyte proliferation and GFAP expression through activation of ERK- and JNK-dependent pathways. ^26^ |

RF=Rosenthal fiber; GFAP=glial fibrillary acidic protein; AxD=Alexander disease; MLC=megalencephalic leukoencephalopathy with subcortical cysts; NPC=neural progenitor cell.


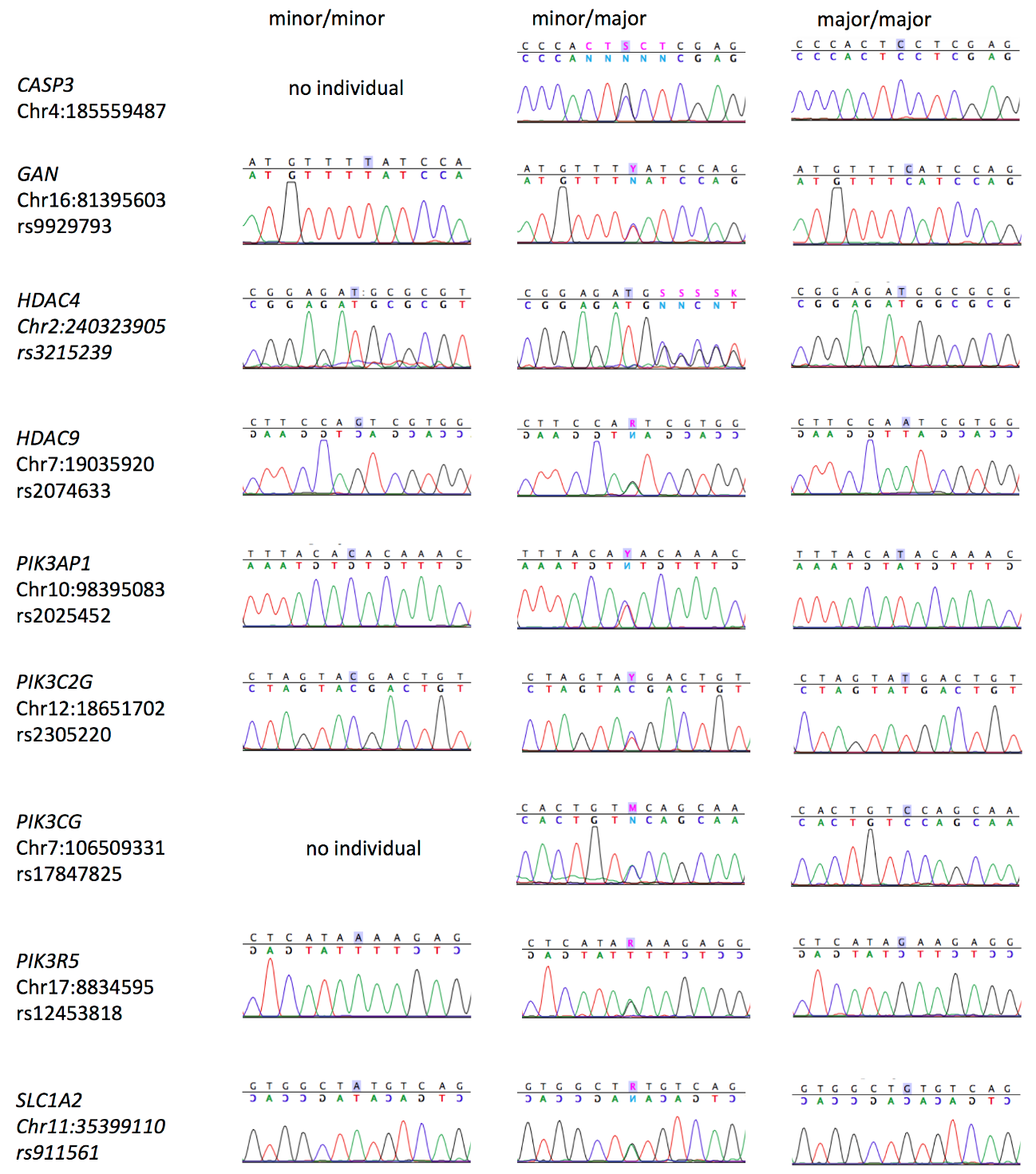


Fig S6-1. Validation of genotyping of 9 SNPs by Sanger method.

Representative chromatogram of each genotype is shown.


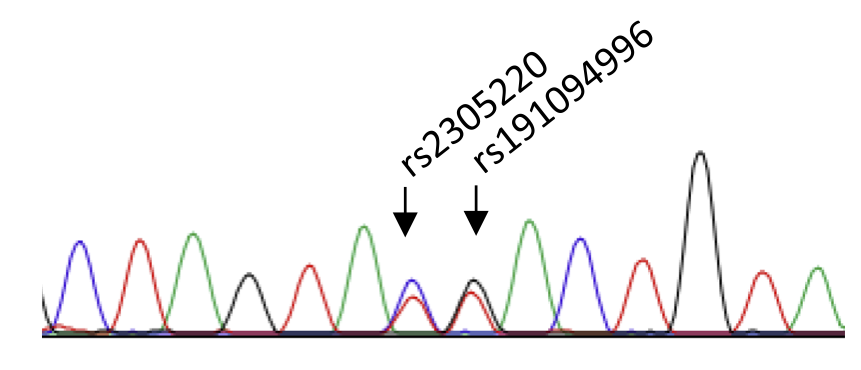


Fig S6-2. Chromatogram of the patient with rs2305220 genotype discrepancy between microarray and Sanger sequence.

Microarray calling was not validated in one genotyping data. In this case, PIK3C2G rs2305220 was called TT by microarray. However, Sanger sequencing revealed that TC is correct and that another SNP rs191094996, which locates next to rs2305220, was heterozygous.

Table S7. Predicted pathogenicity of *GFAP* mutations by multiple tools and literature.

| GFAP mutation | PROVEAN | PolyPhen-2 | SIFT | Mutation Assessor | CADD | Predicted pathogenicity based on the protein structure or stability^27,28^ |
| --- | --- | --- | --- | --- | --- | --- |
| R70W | 2 | 2 | 2 | 2 | 24.1 | Head domain, may affect head to tail assembly to form tetramer; Calpain site, may affect proteolysis |
| M74T | 1 | 1 | 2 | 1 | 22.4 | Rod 1A domain, may affect stability of coiled-coiled dimer |
| R79H | 2 | 2 | 2 | 2 | 27 | Rod 1A domain, may affect stability of coiled-coiled dimer; Caspase site, may affect proteolysis |
| N102K | 1 | 1 | 1 | 1 | 21.9 | Rod 1A domain, may affect stability of coiled-coiled dimer |
| L123P | 2 | 2 | 2 | 2 | 24.5 | Rod 1B domain, may affect stability of coiled-coiled dimer |
| R124_L125insE | 2 | NA | NA | NA | 16.26 | Rod 1B domain, may affect stability of coiled-coiled dimer |
| R126_L127dup | 2 | NA | NA | NA | 16.28 | Rod 1B domain, may affect stability of coiled-coiled dimer. |
| E210K | 2 | 2 | 2 | 2 | 33 | Rod 1B domain, may affect stability of coiled-coiled dimer |
| Y242N | 2 | 2 | 2 | 2 | 25.2 | Rod 2A domain, may affect stability of coiled-coiled dimer |
| E243dup | 2 | NA | NA | NA | 17.97 | Rod 2A domain, may affect stability of coiled-coiled dimer |
| A244V | 1 | 2 | 1 | 1 | 22.1 | Rod 2A domain, may affect stability of coiled-coiled dimer |
| R258C | 2 | 2 | 2 | 2 | 24.5 | Rod 2B domain, may affect stability of coiled-coiled dimer |
| R258H | 2 | 2 | 2 | 1 | 25.2 | Rod 2B domain, may affect stability of coiled-coiled dimer |
| A268D | 2 | 2 | 2 | 2 | 25.8 | Rod 2B domain, may affect stability of coiled-coiled dimer |
| R276L | 2 | 2 | 2 | 2 | 32 | Rod 2B domain, may affect stability of coiled-coiled dimer |
| G301D | 2 | 2 | 2 | 2 | 25.5 | Rod 2B domain, may affect stability of coiled-coiled dimer |
| L357P | 2 | 2 | 2 | 2 | 32 | Rod 2B domain, may affect stability of coiled-coiled dimer |
| D360N | 2 | 2 | 2 | 2 | 32 | Rod 2B domain, may affect stability of coiled-coiled dimer |
| E362G | 2 | 2 | 2 | 2 | 33 | Rod 2B domain, may affect stability of coiled-coiled dimer |
| N386S | 1 | 1 | 1 | 1 | 17.97 | Tail domain, may affect head to tail assembly to form tetramer |
| R416W | 2 | 2 | 2 | 2 | 18.08 | Tail domain, may affect head to tail assembly to form tetramer; Citrullination site, may alter protein conformation |

“1” (green) represents not pathogenic and “2” (red) does pathogenic (PROVEAN (1: neutral, 2: deleterious), PolyPhen-2 (1: benign, 2: possibly and probably damaging), SIFT (1: tolerated, 2: damaging), Mutation Assessor (1: neutral and low, 2: medium and high)). Scaled C-scores are shown in the CADD column and cutoff score 23 is used (< 23: not pathogenic (green), ≥ 23: pathogenic (red)).

NA=not available.

Table S8. Primers used in validation by Sanger sequencing.

| Gene | Position | rs ID | Forward primer | Reverse primer |
| --- | --- | --- | --- | --- |
| *CASP3* | Chr4:185559487 | nd | TAAAGGTATCCATGGAGAACACTG | TGTTAAAGACGAGGTTCAGAGAAG |
| *GAN* | Chr16:81395603 | rs9929793 | ATGAAGTCTTGCTGTGTTGCCTC | AGGAATACCCAGATCTGAGGCATC |
| *HDAC4* | Chr2:240323905 | rs3215239 | CCTAAGCATTTCTTGCAATGACCA | TCTTTCCAACAGGAGTATGACAAC |
| *HDAC9* | Chr7:19035920 | rs2074633 | AATTCTGAACAGCAGCTTCACTTG | GCTGATCAGAATGTCAGGAATGAC |
| *PIK3AP1* | Chr10:98395083 | rs2025452 | GCATCTGCTGTTCCTGGTCTACA | CCAAGAGGGACCCAAGCATCAG |
| *PIK3C2G* | Chr12:18651702 | rs2305220 | CATGGATCCTCACTGCCTGAG | GGAAAGCATTAGGTCACTTACGTG |
| *PIK3CG* | Chr7:106509331 | rs17847825 | CAACAAGTCCTTTGCCAAAGGAGA | CACATGTGGAGGACGTATTCTCCA |
| *PIK3R5* | Chr17:8834595 | rs12453818 | AGTGTCTATAAACAGTTGCTTCAG | TTACCATTGGAGCAAACTGAG |
| *SLC1A2* | Chr11:35399110 | rs911561 | TTCAGGCCTATCTTTGCAGCTTAC | TGCACTGTGTAACAGATGCTCTAC |

Chr=chromosome; nd=not determined.

**Supplementary References**

1 Messing, A. & Brenner, M. in *Neuroglia* (eds Helmut Kettenmann & Bruce R. Ransom) 884-895 (Oxford University Press, 2013).

2 Tian, R., Gregor, M., Wiche, G. & Goldman, J. E. Plectin regulates the organization of glial fibrillary acidic protein in Alexander disease. *Am. J. Pathol.* **168**, 888-897 (2006).

3 Hagemann, T. L., Boelens, W. C., Wawrousek, E. F. & Messing, A. Suppression of GFAP toxicity by alphaB-crystallin in mouse models of Alexander disease. *Hum. Mol. Genet.* **18**, 1190-1199 (2009).

4 Tian, R. *et al.* Alexander disease mutant glial fibrillary acidic protein compromises glutamate transport in astrocytes. *J. Neuropathol. Exp. Neurol.* **69**, 335-345 (2010).

5 Minkel, H. R., Anwer, T. Z., Arps, K. M., Brenner, M. & Olsen, M. L. Elevated GFAP induces astrocyte dysfunction in caudal brain regions: A potential mechanism for hindbrain involved symptoms in type II Alexander disease. *Glia* **63**, 2285-2297 (2015).

6 Sosunov, A. A., Guilfoyle, E., Wu, X., McKhann, G. M., 2nd & Goldman, J. E. Phenotypic conversions of "protoplasmic" to "reactive" astrocytes in Alexander disease. *J. Neurosci.* **33**, 7439-7450 (2013).

7 Hagemann, T. L. *et al.* Gene expression analysis in mice with elevated glial fibrillary acidic protein and Rosenthal fibers reveals a stress response followed by glial activation and neuronal dysfunction. *Hum. Mol. Genet.* **14**, 2443-2458 (2005).

8 LaPash Daniels, C. M. *et al.* Beneficial effects of Nrf2 overexpression in a mouse model of Alexander disease. *J. Neurosci.* **32**, 10507-10515 (2012).

9 Gomez-Pinedo, U., Sirerol-Piquer, M. S., Duran-Moreno, M., Garcia-Verdugo, J. M. & Matias-Guiu, J. Alexander Disease Mutations Produce Cells with Coexpression of Glial Fibrillary Acidic Protein and NG2 in Neurosphere Cultures and Inhibit Differentiation into Mature Oligodendrocytes. *Front. Neurol.* **8**, 255 (2017).

10 Lin, N. H. *et al.* The role of gigaxonin in the degradation of the glial-specific intermediate filament protein GFAP. *Mol. Biol. Cell* **27**, 3980-3990 (2016).

11 Olabarria, M., Putilina, M., Riemer, E. C. & Goldman, J. E. Astrocyte pathology in Alexander disease causes a marked inflammatory environment. *Acta Neuropathol.* **130**, 469-486 (2015).

12 Zhao, W. *et al.* Lack of CUL4B leads to increased abundance of GFAP-positive cells that is mediated by PTGDS in mouse brain. *Hum. Mol. Genet.* **24**, 4686-4697 (2015).

13 Kanski, R. *et al.* Histone acetylation in astrocytes suppresses GFAP and stimulates a reorganization of the intermediate filament network. *J. Cell Sci.* **127**, 4368-4380 (2014).

14 Walker, A. K. *et al.* Astrocytic TDP-43 pathology in Alexander disease. *J. Neurosci.* **34**, 6448-6458 (2014).

15 Chen, M. H., Hagemann, T. L., Quinlan, R. A., Messing, A. & Perng, M. D. Caspase cleavage of GFAP produces an assembly-compromised proteolytic fragment that promotes filament aggregation. *ASN Neuro* **5**, e00125 (2013).

16 Snider, N. T., Park, H. & Omary, M. B. A conserved rod domain phosphotyrosine that is targeted by the phosphatase PTP1B promotes keratin 8 protein insolubility and filament organization. *J. Biol. Chem.* **288**, 31329-31337 (2013).

17 Bachetti, T. *et al.* A novel polymorphic AP-1 binding element of the GFAP promoter is associated with different allelic transcriptional activities. *Ann. Hum. Genet.* **74**, 506-515 (2010).

18 Yoshida, T. *et al.* Effects of a polymorphism in the GFAP promoter on the age of onset and ambulatory disability in late-onset Alexander disease. *J. Hum. Genet.* **58**, 635-638 (2013).

19 Melchionda, L. *et al.* Adult-onset Alexander disease, associated with a mutation in an alternative GFAP transcript, may be phenotypically modulated by a non-neutral HDAC6 variant. *Orphanet J. Rare Dis.* **8**, 66 (2013).

20 Pekny, T. *et al.* Synemin is expressed in reactive astrocytes and Rosenthal fibers in Alexander disease. *APMIS* **122**, 76-80 (2014).

21 Herrera, F., Maher, P. & Schubert, D. c-Jun N-terminal kinase controls a negative loop in the regulation of glial fibrillary acidic protein expression by retinoic acid. *Neuroscience* **208**, 143-149 (2012).

22 Tang, G., Xu, Z. & Goldman, J. E. Synergistic effects of the SAPK/JNK and the proteasome pathway on glial fibrillary acidic protein (GFAP) accumulation in Alexander disease. *J. Biol. Chem.* **281**, 38634-38643 (2006).

23 Herrera, F., Chen, Q. & Schubert, D. Synergistic effect of retinoic acid and cytokines on the regulation of glial fibrillary acidic protein expression. *J. Biol. Chem.* **285**, 38915-38922 (2010).

24 Chen, Y. S., Lim, S. C., Chen, M. H., Quinlan, R. A. & Perng, M. D. Alexander disease causing mutations in the C-terminal domain of GFAP are deleterious both to assembly and network formation with the potential to both activate caspase 3 and decrease cell viability. *Exp. Cell Res.* **317**, 2252-2266 (2011).

25 Nielsen, A. L. *et al.* A new splice variant of glial fibrillary acidic protein, GFAP epsilon, interacts with the presenilin proteins. *J. Biol. Chem.* **277**, 29983-29991 (2002).

26 Gadea, A., Schinelli, S. & Gallo, V. Endothelin-1 regulates astrocyte proliferation and reactive gliosis via a JNK/c-Jun signaling pathway. *J. Neurosci.* **28**, 2394-2408 (2008).

27 Kim, B., Kim, S. & Jin, M. S. Crystal structure of the human glial fibrillary acidic protein 1B domain. *Biochem. Biophys. Res. Commun.* **503**, 2899-2905 (2018).

28 Yang, Z. & Wang, K. K. Glial fibrillary acidic protein: from intermediate filament assembly and gliosis to neurobiomarker. *Trends Neurosci.* **38**, 364-374 (2015).
